# Glutamine transporter SLC1A5 inhibits autophagy-mediated CD276 degradation to promote esophageal cancer progression

**DOI:** 10.1080/15384047.2026.2621606

**Published:** 2026-01-28

**Authors:** Chunyan Wang, Hongyan Zhang, Chaonan Guan, Yuying Li, Shengli Yang, Lan Huang

**Affiliations:** a Translational Medicine Center, The First Affiliated Hospital of Zhengzhou University, Zhengzhou, People's Republic of China

**Keywords:** Esophageal squamous cell carcinoma, CD276, glutamine metabolism, autophagy, SLC1A5

## Abstract

**Background:**

CD276/B7-H3 is an immune checkpoint molecule often overexpressed in cancers, representing a potential therapeutic target. The underlying mechanisms for CD276 upregulation remain unclear. This study investigates how glutamine metabolism affects CD276 protein stability and esophageal squamous cell carcinoma (ESCC) progression.

**Methods:**

CD276 and SLC1A5 expression were analyzed in 90 ESCC clinical tissues and TCGA/GEO datasets. CCK-8, colony formation, wound healing and transwell assays were performed in KYSE150 and KYSE450 cells. Autophagy was quantified by immunofluorescence and western blot. Mitochondrial reactive oxygen species (ROS) levels measured by flow cytometry. Rescue experiments used *N*-acetylcysteine (NAC) and chloroquine (CQ). Finally, antitumor effects of SLC1A5 inhibitor V9302 in the presence or absence of CD276 were evaluated in NOD/SCID mice (*n* = 5 per group) bearing KYSE150 xenografts.

**Results:**

CD276 and SLC1A5 upregulated in ESCC tissues (*P* < 0.05). CD276 overexpression enhanced ESCC cell proliferation and migration by 42.3% and 58.7%, respectively (*P* < 0.01). CQ but not MG-132 increased CD276 expression in ESCC cells. SLC1A5 stabilized CD276 protein without altering CD276 mRNA levels, by suppressing ROS-dependent autophagic degradation. NAC reversed ROS-induced CD276 degradation, while CQ abrogated CD276 downregulation upon glutamine metabolism inhibition. Inhibiting glutamine metabolism could reverse ESCC cell proliferation induced by CD276 overexpression. Moreover, combination of V9302 and CD276 knockout significantly reduced KYSE150 cell-derived xenograft tumor volume by 65.2% (95% CI 58.3–72.1%, *P* < 0.001) in NOD/SCID mice, without affecting mouse body weight (*P* > 0.05).

**Conclusion:**

SLC1A5 enhances CD276 stability by suppressing ROS-autophagy signaling, promoting ESCC progression. Targeting glutamine metabolism to enhance CD276 degradation might be a novel therapeutic strategy for ESCC.

## Introduction

Oesophageal cancer is a common digestive malignancy with high global incidence and mortality.^1^ In China, it ranks fifth in cancer prevalence and fourth in cancer-related mortality.^2^ Oesophageal squamous cell carcinoma (ESCC) constitutes over 80% of cases^3^ and is frequently asymptomatic early on, resulting in late diagnosis and five-year survival below 20%.^4^ Current treatments, such as surgery, chemotherapy, and radiotherapy, have limited efficacy and notable side effects, highlighting the need for novel targets and personalised therapies.^5^ Although immunotherapy and targeted therapies show promise, particularly in advanced ESCC,^6^ elucidating the mechanisms of ESCC progression and identifying new molecular targets remain urgent priorities.

CD276 (B7-H3), a member of the B7 ligand family, is upregulated in multiple malignancies and closely linked to tumour growth and metastasis.^7^^,^^8^ CD276 modulates apoptosis to promote cell survival in cervical and gastric cancers,^9^^,^^10^ confers drug resistance to impair therapeutic efficacy in colorectal and ovarian cancers,^11^^,^^12^ and drives angiogenesis to support tumour vascularisation in diverse tumours,^13-15^ highlighting its pro-tumorigenic functions. However, despite these well-characterised roles in regulating core oncogenic processes, the molecular mechanisms driving CD276 upregulation in tumours remain poorly understood.

Metabolic reprogramming is a hallmark of cancer,^16^ and many tumours exhibit glutamine addiction, a critical metabolic adaptation that provides energy, biosynthetic precursors (e.g., nucleotides, amino acids), and redox balance for rapidly proliferating cancer cells.^17^ Nutrient deprivation within the tumour microenvironment, especially glutamine deficiency, can trigger the AMPK-ULK1 signalling pathway, a core cascade driving autophagy initiation.^18^^,^^19^ Autophagy, in turn, has been shown to regulate the stability of oncogenic proteins via lysosomal degradation or ubiquitin-proteasome system (UPS) modulation: for instance, autophagy induction promotes the degradation of membrane-bound or intracellular oncoproteins by facilitating their trafficking to lysosomes or enhancing UPS-mediated ubiquitination.^20^^,^^21^ This glutamine demand is met primarily through upregulated solute carrier transporters on the cell membrane or enhanced activity of key enzymes in glutamine metabolism. SLC1A5, a primary glutamine transporter responsible for mediating glutamine uptake into cancer cells, is frequently overexpressed across tumour types and correlates with poor prognosis,^22^ positioning SLC1A5 as a promising therapeutic target. Notably, whether aberrant glutamine metabolism, particularly SLC1A5-mediated glutamine uptake and subsequent regulation of the autophagy pathway, modulates CD276 expression by altering its protein stability remains unreported.

Building on the knowledge gaps—specifically the uncharacterised upstream metabolic regulators of CD276 and the unclear crosstalk between SLC1A5-mediated glutamine metabolism and oncoprotein stability in ESCC—this study aimed to test the hypothesis that SLC1A5-mediated glutamine transport stabilises CD276 protein, thereby promoting the aggressive phenotype of ESCC cells. Elucidating this connection is of paramount importance, as it could reveal novel strategies to therapeutically target CD276 by exploiting the metabolic vulnerabilities of ESCC.

In this study, our results suggest that glutamine metabolism markedly influences CD276 protein expression and confirmed that high SLC1A5 expression promotes CD276 stability in ESCC by suppressing ROS-dependent autophagic–lysosomal degradation. Targeting glutamine metabolism to enhance CD276 degradation may thus offer a novel therapeutic strategy for ESCC.

## Materials and methods

### Bioinformatics analysis

CD276 and SLC1A5 expression was analysed TCGA-ESCA dataset (https://portal.gdc.cancer.gov/projects/TCGA-ESCA) via UCSC Xena platform (Version 1.41.0; last accessed: October 15, 2024). with data normalised by TCGA's standard FPKM normalisation and batch effects corrected using the Combat pipeline. Validation was performed in an ESCC dataset (GSE225178) from the Gene Expression Omnibus (GEO; https://www.ncbi.nlm.nih.gov/geo/query/acc.cgi?acc=GSE225178) using GEO2R (Build 2024-08-12)—a web-based tool integrated with NCBI GEO, which utilises R version 4.4.1 and Bioconductor version 3.18, applying quantile normalisation (GEO2R default). Tumour and normal samples were distinguished based on the clinical metadata provided by the TCGA and GEO databases, with no significant differences in age/gender between groups (*P* > 0.05) for matching. Both datasets used the Benjamini-Hochberg (BH) method for multiple-testing correction (FDR adjustment), with log2 fold change (log2FC) and 95% confidence intervals (CIs) as effect sizes.

### Collection of clinical samples

90 specimens (60 ESCC tissues and 30 patient-matched adjacent normal tissues) were collected from ESCC patients who underwent radical surgical resection at The First Affiliated Hospital of Zhengzhou University (March 2023 and February 2024). Patient selection criteria: Inclusion: Primary ESCC (histopathologically confirmed); age 18–75 y; 8th AJCC TNM Stage I (*n* = 8), II (*n* = 35), III (*n* = 17); histological grade (G1: *n* = 12, G2: *n* = 30, G3: *n* = 18, confirmed by two pathologists); complete clinicopathological data; informed consent. Exclusion: Metastatic ESCC, preoperative neoadjuvant therapy, severe comorbidities, pregnancy/lactation, or incomplete data. All patients received radical surgery alone without preoperative adjuvant therapy. Adjacent normal tissue matching: Adjacent normal tissues were histopathologically confirmed non-tumour tissues, collected ≥5 cm from the tumour margin, and processed simultaneously with matched tumour tissues. Tissues were dissected within 30 minutes of resection, rinsed with ice-cold PBS, fixed in 4% PFA at 4 °C for 24 hours, dehydrated via graded ethanol, cleared in xylene, and embedded in paraffin. Serial 4 μm sections were mounted on poly-L-lysine-coated slides and stored at 4 °C. All 60 ESCC tissues and 30 adjacent normal tissues were used for immunofluorescence staining to assess CD276/SLC1A5 expression rates; 10 ESCC (Stage I: *n* = 2, II: *n* = 5, III: *n* = 3; G1: *n* = 2, G2: *n* = 5, G3: *n* = 3) tissues and 5 adjacent normal tissues were randomly selected for QC validation. Specimen-assay mapping is provided in Supplementary Table S2. The study was approved by the Ethical Committee of The First Affiliated Hospital of Zhengzhou University (Protocol No. 2023-KY-0406-003). Written informed consent was obtained from all patients or their legal guardians, and all procedures complied with the Declaration of Helsinki.

### Immunofluorescence staining

Immunofluorescence staining for CD276 or SLC1A5 was performed on ESCC tissue microarrays (ethics approval: Ethics Committee of The First Affiliated Hospital of Zhengzhou University, No. 2023-KY-0406-003). Slides were dewaxed, rehydrated, and antigen—retrieved with preheated EDTA. After PBS washes, tissues were permeabilized with 0.3% Triton X-100 (20 min) and blocked with 5% bovine serum albumin for 1 h at room temperature. Slides were incubated overnight at 4 °C with primary antibodies: anti-CD276 (#740074T, Thermo Fisher Scientific), anti-SLC1A5 (#8057, Cell Signalling Technology) or anti-LAMP1(#9091S, Cell Signalling Technology) or isotype controls (rabbit IgG for SLC1A5/LAMP1, rat IgG for CD276, same concentration as primary antibodies). Following washes, secondary antibody (Alexa Fluor® 488 goat anti-rabbit IgG (#ab150077, Abcam) for SLC1A5 and LAMP1. Alexa Fluor® 568 donkey anti-rat IgG (#ab175475, Abcam) and Alexa Fluor® 594 goat anti-rat IgG (#ab150160, Abcam) for CD276) were incubated for 1 h at room temperature. Nuclei were counterstained with Hoechst (#C1011, Beyotime) for 15 min. Images were acquired on a Carl Zeiss confocal microscope and quantified using Zen software (Version 3.6). Three non-overlapping regions were selected per tissue core. Otsu segmentation (min area = 50 μm²; circularity = 0.4–1.0), Rolling Ball background correction (radius = 50 pixels), positive signal defined as intensity exceeding 95th percentile of controls. Mean fluorescence intensity (MFI) was recorded. Clinical samples: 60 ESCC + 30 adjacent normal tissues. For cell experiments, all assays were performed with 3 independent biological replicates (from separate passages), each with 3 technical replicates. Quantification was conducted in a double-blinded manner by two investigators, confirming excellent interobserver agreement.

### Cell lines and cell culture

KYSE150, KYSE450, and HEK293T were obtained from the Cell Bank of the Chinese Academy of Sciences (Shanghai, China). All cell lines were authenticated via STR profiling (March–April 2024) with ≥0.98 similarity to reference databases and tested negative for mycoplasma contamination (last test: January 2025) using the MycoAlert™ Kit. Experiments were performed within defined passage ranges: KYSE150 (P25–P35), KYSE450 (P22–P32), HEK293T (P18–P28). KYSE150 and KYSE450 cells were cultured in RPMI-1640 medium (#C11875500BT, Gibco), and HEK293T cells in Dulbecco's Modified Eagle Medium (DMEM, #C11995500BT, Gibco). Both media were supplemented with 10% foetal bovine serum (FBS, #10099141C, Gibco), 100 U/mL penicillin, and 100 µg/mL streptomycin (#15140122, Gibco), and contained 2 mM L-glutamine (base medium component). All cells were incubated at 37 °C in a humidified atmosphere with 5% CO₂. For glutamine starvation, cells were plated in complete RPMI-1640 (2 mM glutamine) for 12 h to allow attachment, then washed twice with phosphate-buffered saline (PBS) and switched to glutamine-free RPMI-1640 medium (#21870092, Gibco) supplemented with 10% non-dialysed FBS, 100 U/mL penicillin, and 100 µg/mL streptomycin. All experiments included 3 independent biological replicates (from separate passages) with 3 technical replicates each.

### Plasmids

The cDNA sequences encoding human CD276 (NM_001024736.2) or SLC1A5 (NM_005628.3) was subcloned into the pCDH-Puro vector (Addgene, #167463) using BamH_I and EcoR_I restriction sites. The mCherry-GFP-LC3II fusion construct was similarly subcloned into pCDH-Puro vector using BamH_I and EcoR_I sites. For short hairpin RNA (shRNA)-mediated knockdown, target-specific shRNAs were cloned into the pLKO.1 vector (Addgene, #8453) using Age_I and EcoR_I restriction sites. For CRISPR/Cas9-mediated knockout, single-guide RNAs (sgRNAs) were inserted into lentiCRISPRv2 (Addgene, #52961) using BsmBI restriction sites. All oligonucleotide and primer sequences are listed in Supplementary Table 1. All inserts were verified by Sanger sequencing using vector-specific primers, showing 100% sequence identity and correct orientation. Plasmids were digested with corresponding enzymes and analysed by 1% agarose gel electrophoresis, confirming expected fragment sizes. Plasmids functional validation: Lenti virally transduced into KYSE150/KYSE450 cells, with protein/mRNA overexpression confirmed by Western blot and qRT-PCR; mCherry-GFP-LC3II localisation verified by fluorescence microscopy.

### Lentiviral production

HEK293T cells (6 × 10⁶ cells/10 cm dish, seeded 24 hours pre - transfection) were transfected with target plasmids (8 μg) alongside packaging plasmids psPAX.2 (6 μg) and pMD2.G (2 μg) using polyethyleneimine (#C0537, Beyotime) according to the manufacturer's protocol. Lentiviral supernatant was collected 48 hours post-transfection, centrifuged, filter sterilised and concentrated by Lenti-X Concentrator (#631231, TaKaRa), resuspended in 1/10 of the original volume using Opti-MEM (#21870092, Gibco), aliquoted, flash-frozen in liquid nitrogen, and stored at −80 °C until transduction. Viral titre was determined by TCID50 assay (≥1 × 10⁸ TU/mL). Transduction of KYSE150/KYSE450 cells used MOI = 10, without spin-infection. Transduction conditions are described in the experimental procedures for stable cell line selection. All work was performed under Biosafety Level 2 conditions. Waste was decontaminated, and replication-incompetent viral particles were used to ensure safety.

### Generation of stable cell lines

KYSE150 and KYSE450 cells (5 × 10⁴ cells/well) were seeded in 6-well plates 24 hours pre-transduction. Cells were infected with concentrated lentiviruses in the presence of 10 μg/mL polybrene (#C0351, Beyotime) for 24 hours. Puromycin (1 μg/mL, #A610593, Sangon Biotech) selection was performed for 10 d (medium changed every 48 h). The 1 μg/mL concentration was validated as the minimum lethal concentration. Single-cell clones were isolated via limiting dilution (0.5 cells/well in 96-well plates) and expanded, 3 clones/construct validated via Western blot and qRT-PCR. Target expression/knockdown/knockout stability was monitored at passages 5, 10, and 15 post-selections via qRT-PCR and Western blot. All lines maintained consistent efficiency up to P15, with experiments performed within P5–P10 to ensure phenotypic stability. All functional assays used the 3 validated single clones pooled for consistency.

### Western blot analysis

Samples were washed with PBS and lysed in RIPA assay buffer (#R0010, Solarbio) supplemented with PMSF (#P0100-01, Solarbio) and protease inhibitor cocktail (#P6730, Solarbio). Protein concentration was quantified by BCA assay kit (#P0012, Beyotime). 30 μg protein per lane was separated by SDS-PAGE and transferred to PVDF membranes (#IPVH00010, Millipore). Membranes were blocked with 5% non-fat milk, then incubated overnight at 4 °C with primary antibodies (CD276 (#14058, CST, manufacturer-validated), SLC1A5 (#8057, CST, manufacturer-validated), p62 (#88588, CST, manufacturer-validated), LC3II (#3868, CST, manufacturer-validated), and GAPDH (#2118, CST, manufacturer-validated). After washing, HRP-conjugated secondary antibodies (#7074 or #7076, CST) were incubated for 1 hour at room temperature. Bands were visualised via ECL substrate (#34577, Thermo Fisher Scientific) on a Bio-Rad imaging system (exposure time: 1–5 min per target). Band intensities were quantified via ImageJ software, normalised to GAPDH, and expressed as relative fold change compared to controls. All lines maintained consistent efficiency up to P15, with experiments performed within P5–P15 to avoid phenotypic drift. Three biological replicates and technical controls were included per passage.

### Cell viability measurement

Cells were seeded in 96-well plates at 2 × 10³ cells/well, with trypan blue staining for orthogonal cell-count validation during counting. Cell viability was assessed via Cell Counting Kit-8 (CCK-8) assay (#C0005, TargetMol). At each time point (0, 24, 48, 72, and 96 h), 10 μL of CCK-8 reagent was added to each well, followed by incubation at 37 °C for 2 h. Absorbance at 450 nm was measured with a microplate reader (SpectraMax i3x, Molecular Devices, CA, USA). Controls included: blank control (medium only) and negative control (medium +CCK-8 without cells) to eliminate background. The growth curve was generated from raw OD450 values after subtraction of the negative control values. Experiments included 3 biological replicates (separate passages) with 3 technical replicates each.

### Colony formation assays

Cells were plated in 6-well plates at 1 × 10³ cells/well and incubated at 37 °C for 14 d. Colonies were fixed in 4% paraformaldehyde, stained with 0.2% crystal violet, washed with PBS. Images were photographed using a camera, and the bottom of each well in the 6-well plate was cropped as the region of interest for counting. The number of colonies were quantified using ImageJ software. A colony was defined as a discrete cell cluster containing ≥50 cells, with quantification performed via semi-automated analysis: Images were colour-deconvolved, thresholder (minimum area = 100 μm², circularity = 1.0), and colonies were counted with the ‘Analyse Particles’ function, followed by manual verification to correct for false positives/negatives. Colony area distribution was quantified simultaneously, and results are presented as colony number with 95% confidence intervals (CIs). Representative images with 1 mm scale bars, all experiments included 3 independent biological replicates (from separate passages) with 3 technical replicates each.

### Wound healing assays

Cells were seeded in 6-well plates at 5 × 10⁵ cells/well and incubated overnight to form confluent monolayers. Monolayers were scratched with a sterile 200 μL pipette tip (creating a uniform linear wound), and detached cells were removed by PBS washing twice. Cells were incubated in serum-free medium for 24 h, with cell viability assessed via CCK-8 assay (≥95%) to rule out potential cytotoxic effects induced by serum deprivation. Brightfield images were captured at 10× magnification at 0 h and 24 h post-scratch (3 non-overlapping fields per well). Wound area was quantified via ImageJ using a standardised workflow: Images were converted to grayscale, background-smoothed, and wound edges were automatically detected with the ‘Find Edges’ tool (verified manually). Wound areas were outlined using the ‘Freehand Selection’ tool (pixel intensity threshold: 0–120). Migration was calculated as: Wound closure area = initial wound area − residual wound area at 24 h. Data are reported as individual-field values with 95% confidence intervals (CIs). Analyses were single-blinded; experiments included 3 biological replicates (separate passages) with 3 technical replicates each.

### Transwell migration and invasion assays

Transwell inserts (8 μm pore size, #3422, Corning) were used for both assays. All inserts were pre-hydrated with 200 µL serum-free RPMI-1640 medium at 37 °C for 30 minutes prior to use. Migration assays: upper compartment: 5 × 10⁴ cells resuspended in 200 µL serum-free medium were seeded per insert. Lower compartment: 600 µL complete medium supplemented with 20% FBS was added to each well. Invasion assays: matrigel (#356231, Corning) was diluted to 1 mg/mL with serum-free RPMI-1640 medium, and 50 µL of the diluted Matrigel was added to the upper compartment of each insert. Coated inserts were incubated at 37 °C for 1 hour to allow polymerisation. Upper compartment: 8 × 10⁴ cells resuspended in 200 µL serum-free medium were seeded per Matrigel-coated insert. Lower compartment: 600 µL complete medium supplemented with 20% FBS was added to each well. All plates were incubated at 37 °C with 5% CO₂ for 24 hours. After incubation, non-migrated/non-invaded cells on the upper surface of the membrane were gently removed with a cotton swab. Cells on the lower surface were fixed with 4% paraformaldehyde and stained with 0.2% crystal violet. Migrated or invaded cells were observed and photographed under an optical microscope with 3 non-overlapping fields captured per insert. Cell numbers were quantified using ImageJ software via the ‘Analyse Particles’ function. Experiments included 3 biological replicates (with 3 technical duplicates each), and all analyses were performed in a single-blinded manner to avoid bias.

### RNA isolation and quantitative reverse transcription polymerase chain reaction (RT-qPCR)

Total RNA was isolated using TRIzol reagent (#9109, TaKaRa). cDNA was synthesised using PrimeScript™ RT Reagent Kit with gDNA Eraser (#RR047A, TaKaRa). Target gene expression was quantified by qPCR using 2X Universal SYBR Green Master Mix (#B690016, Sangon Biotech). Amplification efficiencies of all primers (Supplementary Table 1) were verified as 90–110% via standard curve analysis, with single melt curves confirmed. Relative mRNA levels were calculated via the 2^−ΔΔCt^ method and normalised to GAPDH. Experiments included 3 biological replicates with 3 technical replicates each.

### Flow cytometry

Surface staining for CD276: Cells were washed with FACS buffer (PBS + 2% FBS), stained with Zombie Violet (#423113, Biolegend) for 10 min at RT for live/dead exclusion (gated on Zombie Violet- cells). Then cells were incubated with APC-conjugated anti-CD276 (#351006, Biolegend, 1:200) or APC-conjugated rat IgG isotype control (#400612, Biolegend, 1:200). APC FMO control was included to define positive thresholds. Cells were analysed on BD FACSCelesta, data processed with FlowJo. Mitochondrial ROS detection: Cells were stained with Zombie Violet for 10 min at RT for live/dead exclusion. Then incubated with 0.5 mL assay buffer at 37 °C in the dark for 30 min, then 1 µL of 500× MitoROS™ 580 (#22970, AAT Bioquest) was added. After a further 1 h incubation at 37 °C, cells were washed with PBS and resuspended in 0.5 mL complete culture medium. Fluorescence was analysed on a BD FACSCelesta and processed with FlowJo. Unstained cells (incubated with assay buffer only, no MitoROS™ 580) served as a negative control. All samples applied standardised gating with full plots in Supplementary Figure S4. Results are presented as Mean Fluorescence Intensity; experiments included 3 biological replicates with 3 technical replicates each.

### Autophagic flux analysis

Autophagic flux was assessed with a mCherry-GFP-LC3II dual-labelled system. Cells were transfected with mCherry-GFP-LC3II lentivirus and incubated for 48 h, with chloroquine (CQ) as the autophagy inhibitor control (bafilomycin was excluded given the functional redundancy of the two inhibitors in blocking autophagic flux). After fixation with ice-cold methanol, nuclei were counterstained with Hoechst (Beyotime). LC3II puncta were imaged on a Carl Zeiss confocal microscope. For quantification, regions of interest (ROI) were defined as individual cells based on Hoechst-stained nuclei; puncta segmentation was performed using ImageJ with a strict threshold criterion for fluorescence intensity (GFP: 50–255, mCherry: 60–255) and puncta size (0.1–2.0 μm²) were added to ensure accurate puncta identification. All analyses were conducted by an investigator blinded to experimental groups. Yellow puncta (mCherry⁺GFP⁺, autophagosomes) and red-only puncta (mCherry⁺GFP⁻, autolysosomes) were counted, with ≥50 cells analysed per group (from ≥5 random fields). Experiments included 3 biological replicates with 3 technical replicates each.

### Animal experiments

All animal experiments were reviewed and approved by the Ethics Committee of Henan Provincial Laboratory Animal Centre (ZZU-LAC20250522[15]) and conducted in compliance with their established guidelines. Five-week-old female NOD/SCID mice were obtained from Vital River Laboratory Animal Technology (Beijing, China) with a total of 40 mice and maintained under specific-pathogen-free conditions. Mice were randomly allocated into four groups (ten mice per group) and injected subcutaneously into the right flank with 5 × 10^6^ KYSE150 cells transduced with empty vector, CD276 overexpression, SgNC, or SgCD276. Seven days after tumour inoculation, drug intervention began once palpable tumours had developed. The treatment group (five mice per group) received intraperitoneal injections of V9302 at 75 mg/kg every 2 d, while the control group (five mice per group) received an equivalent volume of solvent. Tumour dimensions were measured every 2 d with calipers, and tumour volume was calculated as 0.5 × length × width². The experiment was concluded when tumour volume reached approximately 1500 mm³. In accordance with ethical guidelines for animal welfare, the mice were euthanized by cervical dislocation. Subsequently, tumours were excised, photographed, and weighed.

### Data analysis

All data are presented as mean ± standard deviation (SD) with 95% confidence intervals (CIs) to reflect effect precision. Statistical analyses were performed using GraphPad Prism 9.0 (GraphPad Software, San Diego, CA, USA). Exact *P* values are now reported for all comparisons. Two-tailed independent-samples Student's t-test was used for pairwise comparisons; one-way ANOVA was employed for multi-group comparisons. Statistical significance was defined as adjusted *P* < 0.05 for multiple comparisons and unadjusted *P* < 0.05 for pairwise comparisons.

## Results

### CD276 is upregulated in ESCC and promotes the proliferation and metastasis of ESCC cells

**Figure 1.**
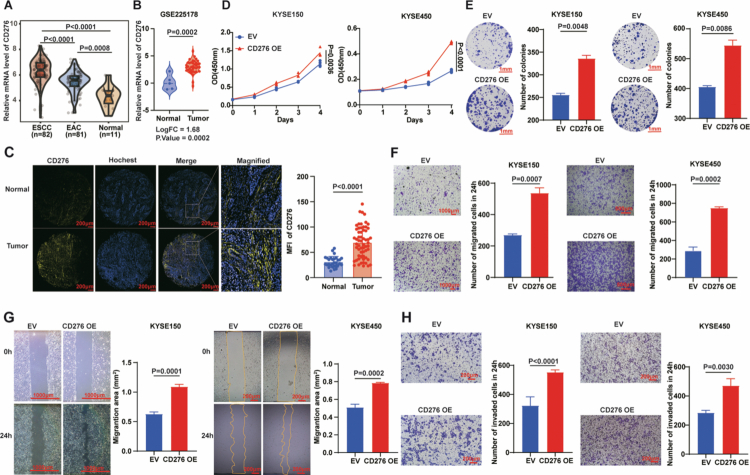
CD276 is upregulated in ESCC and promotes the proliferation and metastasis of ESCC cells. A: CD276 expression was analysed in 11 normal oesophageal tissues, 81 EAC tissues, and 82 ESCC tissues from the TCGA database. B: Violin plot of CD276 mRNA levels in ESCC tissues compared with normal tissues in the GEO database (GSE225178) (|log2(fold change) |≥1, adjusted *P* < 0.05). C: Immunofluorescence staining for CD276 protein levels in a tissue microarray containing 60 ESCC tissues and 30 normal oesophageal tissues. Scale bar = 200 μm. D: Cell viability of CD276 overexpressed (CD276 OE) KYSE150 and KYSE450 cells was measured by CCK-8 assays, with empty vector (EV)-transfected cells as negative controls. E: Colony formation assay and quantitative analysis demonstrated the clonogenic potential of KYSE150 and KYSE450 cells after CD276 overexpression. Scale bar = 1mm. F–H: The effects of CD276 overexpression on cell migration and invasion were evaluated using three approaches: transwell migration assays (Scale bar = 1000 μm (KYSE150), Scale bar = 500 μm (KYSE450)) (F), wound healing assays (Scale bar = 1000 μm (KYSE150), Scale bar = 200 μm (KYSE450)) (G), and Matrigel-based invasion assays (Scale bar = 200 μm) (H). Images were quantified using ImageJ software. *n* = 3 per group. *P* values were calculated using Student's t-test. All quantitative data are presented as mean ± SD with 95% CIs.

Analysis of TCGA data revealed that CD276 expression was significantly higher in ESCC than in oesophageal adenocarcinoma and adjacent normal tissues (Figure 1A). This finding was further validated using the GEO dataset GSE225178 (Figure 1B). In addition, we evaluated the protein levels of CD276 using immunofluorescence staining on a tissue microarray containing 60 primary ESCC specimens and 30 matched normal oesophageal tissues. CD276 protein levels were significantly higher in tumour regions than that in adjacent normal tissues (Figure 1C). Collectively, these results underscore the notable upregulation of CD276 in human ESCC.

To investigate the role of CD276 in ESCC cells, we established stable CD276 overexpressing KYSE150 and KYSE450 cell lines by lentiviral transfection (Figure S1A). We performed CCK-8 assays to explore the impact of CD276 on the growth of ESCC cells. CD276 overexpression significantly increased cell proliferation (Figure 1D). Additionally, colony-formation assays showed that CD276 overexpressing cells formed more colonies (Figure 1E). To further elucidate the contribution of CD276 in the migratory capacity of ESCC cells, we conducted transwell migration and wound healing assays. The results showed the capacity of migratory (Figure 1F) and wound closure (Figure 1G) was enhanced in CD276 overexpression cells. Additionally, Matrigel-coated transwell invasion assays revealed that CD276 overexpression markedly enhanced the invasive capacity of ESCC cells (Figure 1H).

To further confirm the biological role of CD276 in ESCC cells, we knocked out CD276 in KYSE150 and KYSE450 cells using CRISPR/Cas9 technology (Figure S1A). CD276 knockout attenuated cell proliferation, as demonstrated by CCK-8 and colony-formation assays (Figure S1B, C). Transwell migration and wound-healing assays confirmed that CD276 knockout impaired the migratory potential of ESCC cells (Figure S1D, E). Likewise, matrigel invasion assays showed that CD276 knockout suppressed the invasive potential of ESCC cells (Figure S1F). Collectively, our functional analyses demonstrate that elevated CD276 expression promotes proliferation, migration, and invasion of ESCC cells.

Furthermore, re expression of CD276 in CD276 deficient ESCC cells restored the proliferative, migratory, and invasive capacities that were diminished upon CD276 loss (Figure S2A–D). To rule out the possibility that the impaired migration or invasion were secondary to reduced proliferation, we performed short-term assays (8 h) to minimise the potential effects of cell proliferation. Notably, CD276 overexpression still enhanced migration (Figure S2C) and invasion (Figure S2D), confirming that CD276 regulates these processes independently of its role in promoting proliferation. This functional rescue experiment further substantiates the essential role of CD276 in maintaining the aggressive phenotype of ESCC cells.

### The degradation of CD276 protein is regulated by glutamine metabolism in ESCC cells

**Figure 2.**
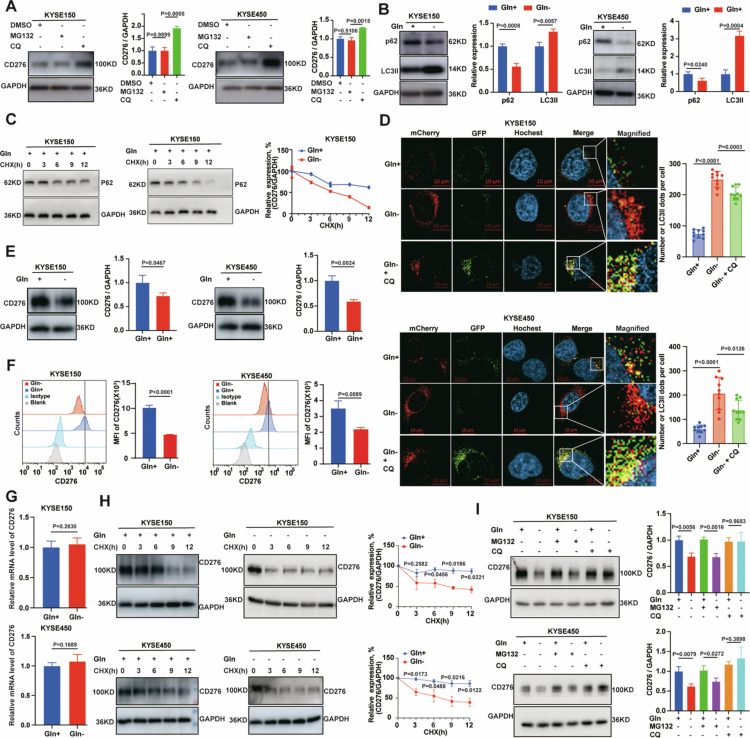
Degradation of CD276 protein is regulated by glutamine metabolism in ESCC cells. A: Western blot and quantitative analysis of CD276 in KYSE150 and KYSE450 cells treated with or without MG132 (10 μM, 12 h) or CQ (25 μM, 12 h). B: Expression of p62 and LC3II in KYSE150 and KYSE450 cells after glutamine deprivation was assessed by western blot and the quantification. C: CHX-chase assay was performed to evaluate P62 protein degradation in KYSE150 cells under glutamine deprivation; and band intensities were quantified by ImageJ and are shown. D: Autophagic flux was measured in KYSE150 and KYSE450 cells infected with mCherry-GFP-LC3II lentivirus under glutamine exist、glutamine deprivation and glutamine deprivation +CQ. Scale bar = 10 μm, *n* = 10. E-F: CD276 expression and quantification results in KYSE150 and KYSE450 cell lines after glutamine deprivation was assessed by western blot (E) and flow cytometry (F). G: CD276 mRNA levels in KYSE150 and KYSE450 cells after 24 h of glutamine deprivation were measured by RT-qPCR. H: CHX-chase assay was performed to evaluate CD276 protein degradation in KYSE150 and KYSE450 cells under glutamine deprivation; and band intensities were quantified by ImageJ and are shown. I: Western blot and quantification of protein bands analysis of CD276 protein levels in KYSE150 and KYSE450 cells under glutamine deprivation with or without MG132 or CQ. *n* = 3 per group. *P* values were calculated using Student's t-test. All quantitative data are presented as mean ± SD with 95% CIs.

The expression level of CD276 protein is modulated not only through transcriptional regulation,^23^ but also via proteolytic degradation mechanisms.^24^ To investigate how CD276 protein is degraded, we treated ESCC cells with the proteasome inhibitor MG132 or the lysosomal inhibitor chloroquine (CQ). Western blot analysis showed CQ, but not MG132, caused the accumulation of CD276 protein levels, indicating that CD276 is predominantly degraded via the autophagy–lysosomal pathway (Figure 2A).

It has been reported that autophagy–lysosomal pathway is highly sensitive to cellular metabolic status and is often activated by nutrient starvation in tumours.^17^^,^^25^ We examined the effect of glutamine deprivation on autophagy and CD276 expression. The results demonstrated glutamine starvation induced autophagy, evidenced by increased LC3II and decreased p62 (Figure 2B). The results demonstrated accelerated degradation of p62 under glutamine deprivation conditions (Figure 2C). mCherry-GFP-LC3II reporter analysis also showed that a marked increase in LC3II-positive puncta in KYSE150 and KYSE450 cells (Figure 2D). To confirm that CD276 degradation depends on autophagic-lysosomal pathway, we performed a co-localisation assay of CD276 and LAMP1. The tumour cells were cultured with glutamine-free medium for 24 h, CD276 and LAMP1 colocalization was observed (Figure S3A). Consistently, glutamine deprivation markedly reduced CD276 protein, as shown by western blot (Figure 2E) and flow cytometry analysis (Figure 2F), whereas RT-qPCR analysis revealed no significant changes in CD276 mRNA levels (Figure 2G). These results indicate that glutamine deprivation regulates CD276 expression via the autophagic-lysosomal pathway rather than through transcriptional mechanisms. To further evaluate the effect of glutamine deprivation on CD276 protein stability, we performed cycloheximide (CHX) chase assays after glutamine deprivation. The results demonstrated accelerated degradation of CD276 under glutamine deprivation conditions (Figure 2H). Finally, glutamine deprivation induced CD276 degradation was reversed by CQ but remained unaffected by MG132 (Figure 2I). In cells cultured with glutamine-free medium in the presence of CQ, the quenched GFP signal recovered, suggesting that CQ blocked autophagosome–lysosomal fusion (Figure 2D). These findings collectively indicate that glutamine deprivation stimulates autophagy–lysosomal degradation of CD276 in ESCC cells.

V9302, a small-molecule antagonist of glutamine transporter SLC1A5, significantly suppressed glutamine metabolism. Loss of SLC1A5 function inhibits glutamine metabolism and activates autophagy.^26^ As expected, the addition of V9302 increased LC3II accumulation and decreased p62 levels (Figure S3B). Quantitative immunofluorescence imaging further demonstrated that V9302 treatment resulted in a significant increase in the number of mCherry-GFP-LC3II puncta (Figure S3C). Moreover, V9302 markedly reduced CD276 protein levels in KYSE150 and KYSE450 cells, as shown by western blot (Figure S3D) and flow cytometry analyses (Figure S3E), whereas RT-qPCR revealed no changes in CD276 mRNA levels (Figure S3F). These data indicate that V9302 regulates CD276 at the protein level rather than transcriptionally. CHX chase assays also showed accelerated degradation of CD276 after V9302 treatment (Figure S3G). Importantly, V9302-induced degradation was rescued by CQ but not by MG132 (Figure S3H). In cells cultured with V9302 in the presence of CQ, the quenched GFP signal recovered, suggesting that CQ blocked autophagosome–lysosomal fusion (Figure S3C). These findings further support that inhibition of glutamine metabolism promotes the autophagy-lysosomal degradation of CD276 in ESCC cells.

### SLC1A5 is upregulated in ESCC and regulates the proliferation and metastasis of ESCC cells

**Figure 3.**
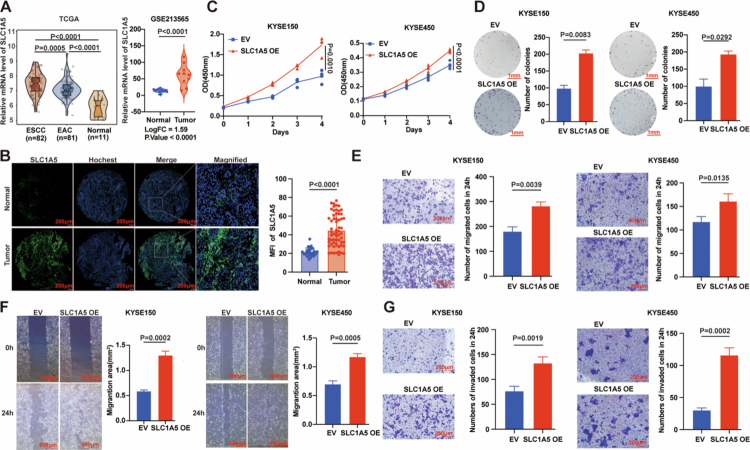
SLC1A5 is upregulated in ESCC and regulates proliferation and metastasis of ESCC cells. A: Violin plots comparing SLC1A5 mRNA expression between TCGA ESCC specimens and EAC specimens or normal tissues (left). Transcriptional expression of SLC1A5 was also analysed in ESCC tissues from the GEO database (GSE213565, right). B: SLC1A5 protein levels were evaluated by immunofluorescence staining on a tissue microarray comprising 60 ESCC and 30 normal oesophageal tissues. Scale bar = 200 μm. C: The effect of SLC1A5 overexpression (SLC1A5 OE) on cell proliferation was quantified by CCK-8 assay. D: Representative images and quantification of colony formation assays in SLC1A5 overexpressing KYSE150 and KYSE450 cells. Scale bar = 1 mm. E: Transwell migration assays were performed to assess migratory capacity after SLC1A5 overexpression; representative micrographs and quantitative data are shown. Scale bar = 200 μm. F: Wound-healing assays were used to evaluate wound closure in SLC1A5 overexpressing KYSE150 and KYSE450 cells. Scale bar = 500 μm. G: Invasive potential conferred by SLC1A5 was assessed by Matrigel-based invasion assays; representative images and quantitative results are presented Scale bar = 200 μm. All images were quantified using ImageJ software. *n* = 3 per group. *P* values were calculated using Student's t-test. All quantitative data are presented as mean ± SD with 95% CIs.

Glutamine metabolic reprogramming is a recognised hallmark of ESCC.^27^ To characterise glutamine metabolism in ESCC cells, we analysed the expression level of SLC1A5. Analysis of TCGA and GEO (GSE213565) datasets revealed markedly elevated SLC1A5 mRNA levels in ESCC compared with normal oesophageal tissues (Figure 3A). Similar results were observed in the protein level in human ESCC specimens (Figure 3B). To evaluate the functional role of SLC1A5, KYSE150 and KYSE450 cells were transfected with lentiviral vectors for SLC1A5 overexpression or knockdown, and efficiency was confirmed by western blot (Figure S5A, S5B). SLC1A5 overexpression significantly increased cell viability (Figure 3C) and colony-forming capacity (Figure 3D). Transwell assays showed enhanced migratory ability (Figure 3E), and wound-healing assays revealed accelerated wound closure (Figure 3F). Matrigel invasion assays further demonstrated a pronounced increase in invasive potential (Figure 3G). Overall, these data indicate that SLC1A5 promotes the ability of proliferation, migration, and invasion of ESCC cells.

Next, we assessed the effects of glutamine metabolism inhibition via SLC1A5 knockdown, V9302 treatment, or glutamine deprivation. SLC1A5 knockdown markedly reduced cell viability (Figure S5C) and colony formation (Figure S5D), and attenuated migration in transwell (Figure S5E) and wound-healing assays (Figure S5F). Matrigel invasion assays showed a significant decrease in invasive capacity (Figure S5G). Likewise, V9302 or glutamine deprivation markedly reduced cell viability (Figure S6A, F), suppressed colony-forming capacity (Figure S6B, G), and impaired both migration (Figure S6C, H) and wound closure (Figure S6D, I). Invasion through Matrigel was also significantly diminished after treatment with V9302 or glutamine deprivation (Figure S6E, J). Collectively, these findings demonstrate that inhibition of glutamine metabolism restrains proliferation and metastatic potential of ESCC cells.

### SLC1A5 overexpression blocks autophagic-lysosomal degradation of CD276 in ESCC cells

**Figure 4.**
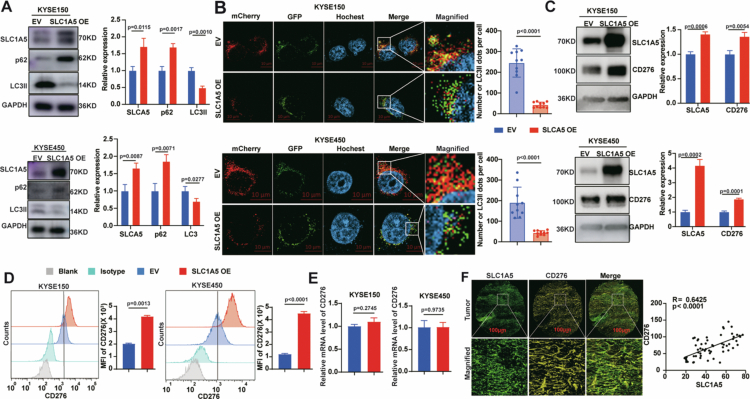
SLC1A5 overexpression blocks autophagic-lysosomal degradation of CD276 in ESCC cells. A: The protein expression levels of autophagy markers p62 and LC3II in SLC1A5-overexpressing KYSE150 and KYSE450 cells. B: Autophagic flux was assessed by infecting SLC1A5-overexpressing KYSE150 and KYSE450 cells with mCherry-GFP-LC3II lentivirus. Cells were imaged by confocal microscopy, and mCherry_(red) and GFP_(green) puncta were quantified. Scale bar = 10 μm. C: Western blot analysis of CD276 and SLC1A5 expression in SLC1A5-overexpressing KYSE150 and KYSE450 cells. Densitometric quantification of CD276 and SLC1A5 band intensity (normalised to GAPDH) from three independent Western blot experiments. D: Flow cytometry quantification of cell-surface CD276 on SLC1A5 overexpressing KYSE150 and KYSE450 cells. E: RT-qPCR was employed to measure CD276 mRNA levels in SLC1A5 overexpressing KYSE150 and KYSE450 cells. F: Representative immunofluorescence staining for SLC1A5 (green) and CD276 (yellow) in 60 ESCC tissues and 30 normal oesophageal tissues. The merged images show positive co-localisation of SLC1A5 and CD276 in tumour sections. Scale bar = 100 μm. *n* = 3 per group. *P* values were calculated using Student's t-test. All quantitative data are presented as mean ± SD with 95% CIs.

To explore whether SLC1A5 upregulation influence the autophagy-mediated CD276 expression, we first analysed the impact of SLC1A5 on autophagy. We found that SLC1A5 overexpression suppressed autophagic activity, as shown by reduced LC3II and elevated p62 levels (Figure 4A). This was corroborated by fewer mCherry-GFP-LC3II puncta, indicating diminished autophagosome formation (Figure 4B). Consistently, SLC1A5 overexpression in KYSE150 and KYSE450 cells markedly increased CD276 protein levels, as confirmed by western blot (Figure 4C) and flow cytometry analysis (Figure 4D), whereas CD276 mRNA remained unchanged (Figure 4E), indicating post-transcriptional regulation. Moreover, in clinical ESCC specimens, SLC1A5 and CD276 expression were positively correlated (Figure 4F).

Conversely, SLC1A5 knockout enhanced autophagy, as evidenced by increased LC3II and decreased p62 (Figure S7A). Quantitative immunofluorescence analysis of mCherry-GFP-LC3II puncta further confirmed the increased formation of autophagosomes in SLC1A5 knockout cells (Figure S7B). SLC1A5 knockout significantly reduced CD276 protein expression (Figure S7C-D) without altering CD276 mRNA levels (Figure S7E). CHX chase assays revealed accelerated CD276 degradation in SLC1A5 knockout ESCC cells (Figure S7F), indicating the essential role of SLC1A5 in maintaining CD276 stability. Crucially, lysosomal inhibition with CQ, but not MG132, restored CD276 levels in SLC1A5 knockout cells (Figure S7G). SLC1A5 knockout cells in the presence of CQ, the quenched GFP signal recovered, suggesting that CQ blocked autophagosome–lysosomal fusion (Figure S7B). Collectively, these findings indicate that SLC1A5 stabilises CD276 by suppressing autophagy in ESCC cells.

### Glutamine metabolism regulates autophagic-lysosomal degradation of CD276 via ROS

**Figure 5.**
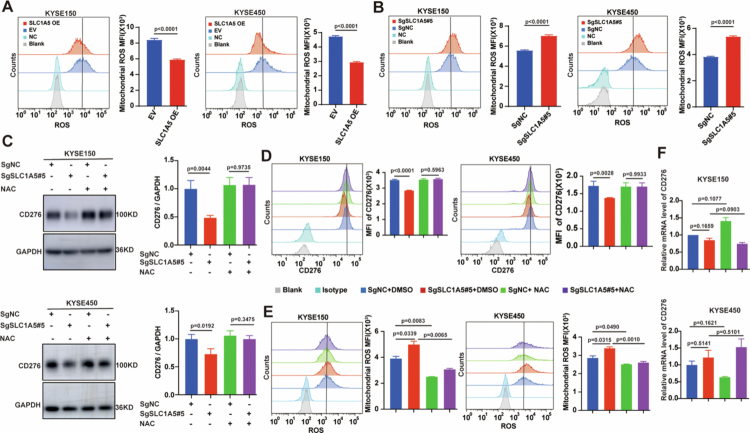
SLC1A5 regulates autophagic-lysosomal degradation of CD276 via ROS. A: Mitochondrial ROS levels were assessed by flow cytometry in SLC1A5 overexpressing KYSE150 and KYSE450 cells. B: Mitochondrial ROS levels were measured using flow cytometry in SLC1A5 knockout KYSE150 and KYSE450 cells. C: CD276 protein levels were examined by western blot in SLC1A5 knockout KYSE150 and KYSE450 cells treated with or without NAC (2.5 mM). Panel on the right shows the respective quantification of Western blot. D: Cell-surface CD276 levels were quantified by flow cytometry in SLC1A5 knockout KYSE150 and KYSE450 cells treated with or without NAC. E: Mitochondrial ROS levels were tracked by flow cytometry in SLC1A5 knockout KYSE150 and KYSE450 models with or without NAC. F: CD276 mRNA expression levels were evaluated by RT-qPCR in SLC1A5 knockout KYSE150 and KYSE450 systems with or without NAC. *n* = 3 per group. *P* values were calculated using Student's t-test. All quantitative data are presented as mean ± SD with 95% CIs.

Previous studies established that nutritional deprivation, hypoxia, ischaemia-reperfusion, and other cellular stressors elevate intracellular ROS levels, thereby triggering autophagy.^28^ We thus explored whether glutamine metabolism controls CD276 degradation in a ROS-dependent manner. Flow cytometry showed that SLC1A5 overexpression significantly reduced mitochondrial ROS in KYSE150 and KYSE450 cells (Figure 5A), whereas SLC1A5 knockout increased mitochondrial ROS (Figure 5B). To test whether the elevated ROS caused CD276 loss, we treated SLC1A5 knockout cells with the cell-permeable scavenger *N*-acetylcysteine (NAC). Western blot revealed that NAC restored CD276 protein levels in SLC1A5-knockout ESCC cells (Figure 5C), and consistent results were obtained by flow cytometry (Figure 5D). Importantly, NAC also reversed the ROS accumulation induced by SLC1A5 knockout (Figure 5E). CD276 mRNA levels remained unchanged after NAC treatment (Figure 5F). Thus, SLC1A5 regulates CD276 stability via ROS at the post-transcriptional level.

We further examined whether direct inhibition of glutamine metabolism leads to ROS-dependent autophagic degradation of CD276. Consistent with SLC1A5 knockout results, flow cytometry analysis showed that glutamine deprivation or the SLC1A5 inhibitor V9302 elevated mitochondrial ROS (Figure S8A-B). Western blot analysis demonstrated that NAC restored CD276 protein levels after glutamine deprivation (Figure S8C) or V9302 treatment (Figure S8D) in ESCC cells. Flow cytometry further confirmed that NAC reversed the downregulation of CD276 induced by glutamine deprivation and V9302 treatment (Figure S8E-F). Notably, NAC reduced elevated ROS following glutamine deprivation or V9302 treatment (Figure S8G-H). After NAC was added, CD276 mRNA levels remained unchanged (Figure S8I-J), indicating that this regulation occurs independently of transcriptional mechanisms. Taken together, these data demonstrate that glutamine inhibition triggers ROS-dependent autophagic degradation of CD276 in ESCC cells.

### Targeting glutamine metabolism mediates autophagic degradation of CD276 to inhibit ESCC progression

Based on the role of glutamine metabolism in regulation of CD276 expression, we investigated whether targeting glutamine metabolism could inhibit CD276 mediated ESCC progression by CCK-8 assays. Our results demonstrated that SLC1A5 overexpression rescued proliferation suppressed by CD276 knockout in KYSE150 and KYSE450 cells (Figure S9A). Conversely, knockout of SLC1A5 significantly inhibited the cell proliferation induced by CD276 overexpression in KYSE150 and KYSE450 cell lines (Figure S9B). Inhibition of glutamine metabolism by V9302 treatment (Figure S9C) or by glutamine deprivation (Figure S9D) effectively rescued the increased proliferation induced by CD276 overexpression in ESCC cells. Furthermore, we investigated whether dual inhibition of glutamine metabolism and CD276 could achieve a better antitumor effect against ESCC. As expected, the combination of CD276 knockout with either SLC1A5 knockout (Figure S9E), V9302 treatment (Figure S9F), or glutamine deprivation (Figure S9G) further suppressed proliferation in vitro. Taken together, these findings suggest that dual inhibition of glutamine metabolism and CD276 represents a promising therapeutic strategy for inhibiting ESCC proliferation.

**Figure 6.**
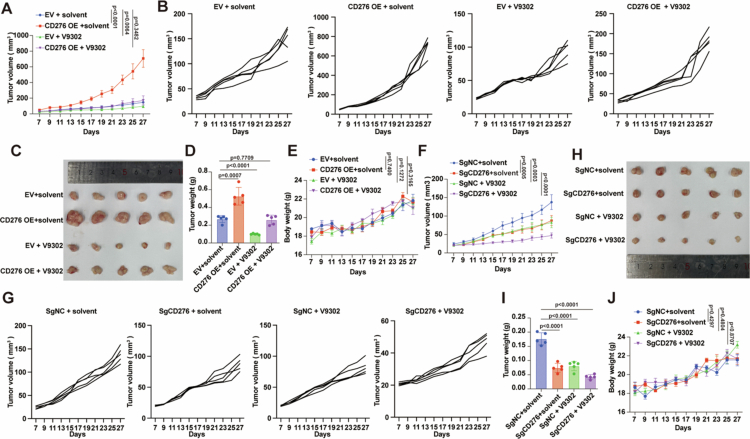
Combined glutamine metabolism inhibition and CD276 blockade suppresses ESCC proliferation *in vivo*. V9302 treatment, combined with either CD276 overexpression or knockout, regulates KYSE150 xenograft tumour growth in NOD/SCID mice. A: Tumour volumes were measured every 48 h until sacrifice on day 27 post-inoculation. Groups: EV + solvent, CD276 OE + solvent, EV + V9302, CD276 OE + V9302. B: Representative individual tumour growth curves from the model in (A). C: Representative images of excised xenograft tumours from the groups in (A). D: Tumour weights from the groups in (A). E: Individual mouse body weights were measured every 2 d for the groups in (A). Representative tumour volumes (F) and individual tumour volume curves (G) in CD276 knockout groups: SgNC + solvent, SgCD276 + solvent, SgNC + V9302, SgCD276 + V9302. Volumes were assessed every 48 h. H: Photographs of harvested tumours corresponding to the groups in (F). I: Tumour weights for the groups in (F). J: Body weights were monitored every 2 d for the groups in (F). Randomisation and blinded assessment were applied for tumour measurement and data analysis. *P* values were calculated using Student's t-test. All quantitative data are presented as mean ± SD with 95% CIs.

To confirm the antitumor effect of targeting glutamine metabolism in vivo, the inhibitory role of V9302 was tested in various KYSE150 xenograft models, including those with empty vector, CD276 overexpression, SgNC, or SgCD276. Seven days after subcutaneous tumour inoculation, the mice were randomised to receive either intraperitoneal V9302 or an equivalent volume of solvent. Consistent with in vitro findings, CD276 overexpressing xenografts exhibited significantly enhanced proliferative capacity compared with those in the empty vector group, as evidenced by increased tumour size (Figure 6A–C) and tumour weight (Figure 6D). V9302 treatment effectively inhibited CD276 driven tumour growth in these models (Figure 6A–D). Conversely, CD276 knockout xenografts showed significant tumour suppression with delayed progression compared to SgNC group, as evidenced by reduced tumour size (Figure 6F–H) and tumour weight (Figure 6I). V9302 treatment further reduced tumour proliferation in CD276 knockout or SgNC group (Figure 6F–H). Critically, these tumour-modulatory effects occurred without systemic toxicity, as indicated by unchanged body weights (Figure 6E, J). Western blot analysis showed a significant decrease in CD276 protein levels in tumour tissues after V9302 administration (Figure S10A–C, E–G). In contrast, qRT-PCR assays revealed that the mRNA level of CD276 remained unchanged under the same treatment conditions (Figure S10D, H). These results collectively indicate that V9302 may regulate CD276 expression at the post-transcriptional or post-translational level, rather than at the transcriptional level. Our findings underscore the cooperative roles of CD276 and glutamine metabolism in ESCC pathogenesis and validate combined targeting of SLC1A5 and CD276 as a novel therapeutic approach.

## Discussion

CD276 promotes tumour progression through cell proliferation, angiogenesis, migration, invasion, and metabolic reprogramming,^29^^,^^30^ though the mechanisms for its upregulation remains unclear. In this study, we observed that CD276 is highly expressed in ESCC and positively associated with SLC1A5 expression. Mechanistically, our preclinical data demonstrate that SLC1A5 maintains CD276 expression by inhibiting ROS-dependent autophagy in ESCC cell lines and xenograft models. Our findings uncover a previously unknown role for SLC1A5 in driving CD276 overexpression and ESCC tumorigenesis in preclinical settings, supporting the potential of jointly targeting CD276 and SLC1A5 as a preclinical therapeutic strategy worthy of further clinical exploration.

We confirmed that CD276 is overexpressed in ESCC tissues compared to adjacent normal tissues, consistent with reports in other cancers.^29^^,^^31^ Notably, recent studies have shown that CD276 expression is associated with advanced TNM stage and poor prognosis in ESCC patients.^32^^,^^33^ Functionally, CD276 overexpression enhanced malignant phenotypes, whereas knockout of CD276 suppressed these malignant behaviours in ESCC cell lines, aligning with its known roles in promoting proliferation, invasion, and metastasis in other cancer types.^34^ Collectively, these data suggest that CD276 plays a significant role in ESCC progression in preclinical models. The molecular mechanisms by which CD276 exerts its oncogenic effects have been partially elucidated in other cancer types, such as promoting migration and invasion via regulating the actin cytoskeleton and RhoA/ROCK1/LIMK1 signalling pathway in colorectal cancer^35^ or promoting VEGFA-mediated angiogenesis via NF-κB pathway activation.^36^ CD276 also confers paclitaxel resistance in breast cancer cells by interfering with the Jak2–Stat3 signalling pathway.^37^ Despite this functional diversity, the reasons for its specific overexpression in ESCC remain unclear and require further investigation in clinical cohorts.

CD276 expression is regulated at transcriptional and post-transcriptional levels: transcriptionally through PRMT5-ALKBH5-mediated m6A modification,^23^ and post-transcriptionally via autophagy-lysosomal degradation in various cancers.^24^^,^^38-40^ In this study, we found that in ESCC cell lines and xenografts, elevated SLC1A5 is associated with reduced lysosomal degradation of CD276. Glutamine metabolic disruption induces autophagic-lysosomal degradation of CD276 without affecting its mRNA in ESCC cells, indicating post-transcriptional control - a finding specific to ESCC that extends prior observations in other cancer types. This observation links CD276 stability to glutamine metabolism and the glutamine transporter SLC1A5 in preclinical ESCC models. Furthermore, we reported that SLC1A5 is upregulated in ESCC tissues (compared to normal tissues) and promotes tumour growth and metastasis in cell lines and xenografts; inhibition of SLC1A5 restricts malignancy in these preclinical models. This observation links CD276 stability to glutamine metabolism and the glutamine transporter SLC1A5 in preclinical ESCC models. Furthermore, we reported that SLC1A5 is upregulated in ESCC tissues (compared to normal tissues) and promotes tumour growth and metastasis in cell lines and xenografts; inhibition of SLC1A5 restricts malignancy in these preclinical models. Recent ESCC-specific studies have shown that SLC1A5-mediated glutamine metabolism supports tumour cell proliferation,^41^^,^^42^ which aligns with our findings. Beyond ESCC, tumour cells compete for glutamine in the microenvironment to facilitate tumour progression.^43^ Such as SLC1A5 upregulation enabling liver cancer cells to outcompete myeloid cells for glutamine ^44^ or suppressing autophagy in head and neck squamous cell carcinoma.^45^ Overall, our preclinical data support a model wherein SLC1A5-dependent glutamine metabolism regulates CD276 stability through autophagy, yet the precise molecular link between glutamine availability and autophagic regulation remains a working hypothesis requiring further validation.

In our experiments, SLC1A5 overexpression decreased LC3II and increased p62 (markers of autophagic flux suppression) in ESCC cells, while SLC1A5 knockout had the opposite effect—a direct observation confirming SLC1A5 inhibits autophagy in ESCC models. This is consistent with reports in fibrotic lung fibroblasts.^46^ Our clinical cohort revealed a strong positive correlation between SLC1A5 and CD276 expression, underscoring their potential clinical relevance. Mechanistically, we directly demonstrated that SLC1A5 inhibition increases ROS in ESCC cells, consistent with reports in pancreatic cancer^47^ and T cells,^48^ and the ROS scavenger NAC reversed CD276 reduction in SLC1A5 knockout cells—a direct link between ROS and CD276 stability in ESCC. Since glutamine restriction activates ROS-dependent autophagy,^49^ and autophagy-ROS interplay is well-established,^50^ we propose that SLC1A5 stabilises CD276 by inhibiting ROS-mediated autophagic degradation in ESCC. However, alternative routes cannot be excluded: for example, SLC1A5 may regulate CD276 stability via glutamine-dependent metabolic intermediates (e.g., *α*-ketoglutarate) that modulate autophagy-related enzymes,^51^ or through indirect regulation of other autophagy receptors (beyond p62) that target CD276 for degradation.^52^ These alternative pathways remain speculative and require future investigation.

Targeted therapies enable precise tumour cell elimination while inhibiting progression and improving survival. The SLC1A5 inhibitor V9302 shows strong antitumor effects across multiple preclinical models,^53^^,^^54^ but faces clinical challenges including poor pharmacokinetics and off-target toxicity.^55^ Meanwhile, CD276-directed CAR-T cells have shown promise in early clinical studies but raise safety concerns such as on-target/off-tumour effects.^56-58^ In our ESCC xenograft models, CD276 overexpression accelerated tumour growth, an effect reversed by V9302, and dual targeting of CD276 and SLC1A5 yielded superior tumour suppression compared to monotherapies. To translate these preclinical findings into a realistic clinical path, we should focus on optimising the therapeutic window through precise dose-finding and pharmacokinetic analyses in xenografts. We also advocate for evaluating combinations with anti-CD276 monoclonal antibodies rather than CAR-T cells to minimise toxicity. Crucially, as glutamine is vital for immune effector function,^59^ validation in immunocompetent models is mandatory. This will clarify the net impact of SLC1A5 inhibition on the tumour microenvironment and verify the safety-efficacy balance of the proposed combination therapy.^60^

While this study reveals how SLC1A5 promotes tumorigenesis by inhibiting CD276 degradation in preclinical models, several limitations should be acknowledged. First, although we observed a positive correlation between SLC1A5 and CD276 in clinical ESCC tissues, this is an associative finding that does not establish causation in humans. Second, our in vitro and xenograft models clarified the core mechanism, further validation using patient-derived organoids or xenografts would strengthen clinical relevance. Third, CD276 is an immunoregulatory protein that suppresses T-cell activation and adaptive immunity.^61^ Our mechanistic findings are based on ESCC cell lines and immunodeficient xenografts, which lack a functional immune system—thus, the interplay between SLC1A5, CD276, and the tumour microenvironment (especially immune cells) remains unclear. To address these limitations, future studies should prioritise validating these findings in patient-derived organoids (PDOs) to better capture clinical heterogeneity. Furthermore, expanding correlation analyses to larger, annotated clinical cohorts with long-term follow-up is essential to establish whether SLC1A5/CD276 co-expression serves as a reliable prognostic or predictive biomarker. Crucially, the use of immunocompetent models will be requisite to fully dissect how SLC1A5 regulates immune cell function within the ESCC microenvironment.

## Conclusion

Our study identifies a novel preclinical mechanism in ESCC whereby SLC1A5 upregulation stabilises CD276 by inhibiting ROS-induced autophagy. This finding provides new insights into the molecular basis of CD276 overexpression in ESCC and supports SLC1A5 as a potential promoter of ESCC progression. Targeting SLC1A5 to trigger autophagic CD276 degradation offers a rational preclinical therapeutic strategy for ESCC. A critical next step will be to validate the SLC1A5-ROS-autophagy-CD276 axis in larger clinical cohorts and explore its relevance in other cancer types co-expressing SLC1A5 and CD276 through translational studies.

## Supplementary Material

Supplementary material Supplemental material clear.

Supplementary Material ARRIVE_guidelines_checklist

## Data Availability

Data used to support the findings of this study are available from the corresponding author upon request.
