# Supplementary material for: Glutamine transporter SLC1A5 inhibits autophagy-mediated CD276 degradation to promote esophageal cancer progression
Source: Cancer Biol Ther. 2026 Jan 28;27(1):2621606. doi: 10.1080/15384047.2026.2621606 (PMC12867364; doi:10.1080/15384047.2026.2621606)
Supplement: Supplementary material — Supplemental material clear. [file KCBT_A_2621606_SM7184.docx]

**Table S1. Sequences for primers**

| **Name** | **Sequence** |
| --- | --- |
| ShNC | CAACAAGATGAAGAGCACCAA |
| ShSLC1A5#2 | GCCTGAGTTGATACAAGTGAA |
| ShSLC1A5#3 | CTGGATTATGAGGAATGGATA |
| SgNC-forward | CACCGGCACTACCAGAGCTAACTCA |
| SgNC-reverse | AAACTGAGTTAGCTCTGGTAGTGCC |
| SgSLC1A5#4-Forward | CACCGCGAGCCCCTTGGAGTCTCG |
| SgSLC1A5#4-Reverse | AAACCGAGACTCCAAGGGGCTCGC |
| SgSLC1A5#5-Forward | CACCGTGGCTGGTAACCGCTACTCC |
| SgSLC1A5#5-Reverse | AAACGGAGTAGCGGTTACCAGCCAC |
| SgCD276#1-Forward | CACCGAGGAAGATGCTGCGTCGGCG |
| SgCD276#1-Reverse | AAACCGCCGACGCAGCATCTTCCTC |
| SgCD276#2-Forward | CACCGACAGGAAGATGCTGCGTCGG |
| SgCD276#2-Reverse | AAACCCGACGCAGCATCTTCCTGTC |
| SgCD276#3-Forward | CACCGCTCACAGGAAGATGCTGCGT |
| SgCD276#3-Reverse | AAACACGCAGCATCTTCCTGTGAGC |
| GAPDH-Forward | GGAGTCAACGGATTTGGTCGTA |
| GAPDH-Reverse | GGCAACAATATCCACTTTACCAGAGT |
| SLC1A5 - Forward | GTGTCCTCACTCTGGCCATC |
| SLC1A5 -Reverse | TACAGGACCGGTCGACTAGC |
| CD276 - Forward | CTGGCTTTCGTGTGCTGGAGAA |
| CD276 - Reverse | GCTGTCAGAGTGTTTCAGAGGC |

**Table S2：ESCC tissue microarray layout**

| ESCC36T | ESCC32T | ESCC26T | ESCC21T | ESCC17T | ESCC12T | ESCC07T | ESCC01T |
| --- | --- | --- | --- | --- | --- | --- | --- |
| ESCC36T | ESCC32T | ESCC26T | ESCC21T | ESCC17T | ESCC12T | ESCC07T | ESCC01T |
| ESCC36N | ESCC32N | ESCC26N | ESCC21N | ESCC17N | ESCC12N | ESCC07N | ESCC01N |
| ESCC27T | ESCC33T | ESCC28T | ESCC22T | ESCC18T | ESCC13T | ESCC08T | ESCC02T |
| ESCC27T | ESCC33T | ESCC28T | ESCC22T | ESCC18T | ESCC13T | ESCC08T | ESCC02T |
| ESCC27N | ESCC33N | ESCC28N | ESCC22N | ESCC18N | ESCC13N | ESCC08N | ESCC02N |
|  | ESCC34T | ESCC29T | ESCC23T | ESCC19T | ESCC15T | ESCC09T | ESCC03T |
|  | ESCC34T | ESCC29T | ESCC23T | ESCC19T | ESCC15T | ESCC09T | ESCC03T |
|  | ESCC34N | ESCC29N | ESCC23N | ESCC19N | ESCC15N | ESCC09N | ESCC03N |
|  | ESCC35T | ESCC31T | ESCC25T | ESCC20T | ESCC16T | ESCC10T | ESCC06T |
|  | ESCC35T | ESCC31T | ESCC25T | ESCC20T | ESCC16T | ESCC10T | ESCC06T |
|  | ESCC35N | ESCC31N | ESCC25N | ESCC20N | ESCC16N | ESCC10N | ESCC06N |

**Supplementary Figure**


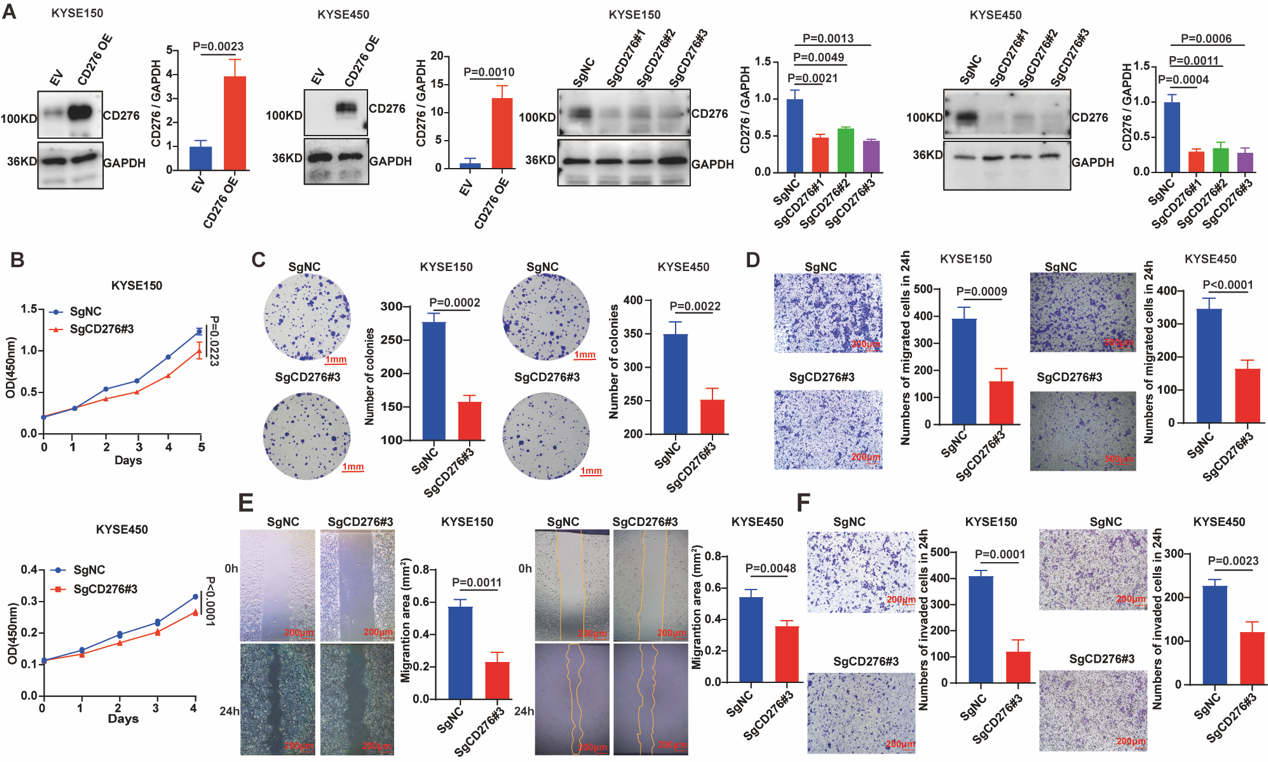


**Figure S1. CD276 knockout inhibits ESCC proliferation and metastasis *in vitro*.**

**A:** Western blot analysis confirmed the ​​efficiency of CD276 overexpression and knockout​​ in KYSE150 and KYSE450 cells. Band intensities were quantified using ImageJ and normalized to GAPDH. **B, C:** Cell proliferation was assessed by CCK-8 assay (**B**) and colony formation assay (Scale bar = 1mm) (**C**). SgNC transfected cells as negative controls. **D:** Transwell migration assays were used to evaluate the migratory capacity of KYSE150 (Scale bar = 200μm) and KYSE450 (Scale bar = 500μm) cells after CD276 knockout (SgCD276). **E:** Wound closure in CD276 knockout KYSE150 and KYSE450 cells was assessed by wound healing assays. (Scale bar = 200μm). **F:** Matrigel-coated transwell invasion assays were used to evaluate changes in invasive activity following CD276 knockout. (Scale bar = 200μm). Images were quantitatively analyzed using ImageJ software. n = 3 per group. P values were calculated using Student’s t-test. All quantitative data are presented as mean ± SD with 95% CIs.

**Figure S2. Re expression of CD276 rescues the impaired proliferation, migration, and invasion induced by CD276 knockout in ESCC cells.**

**
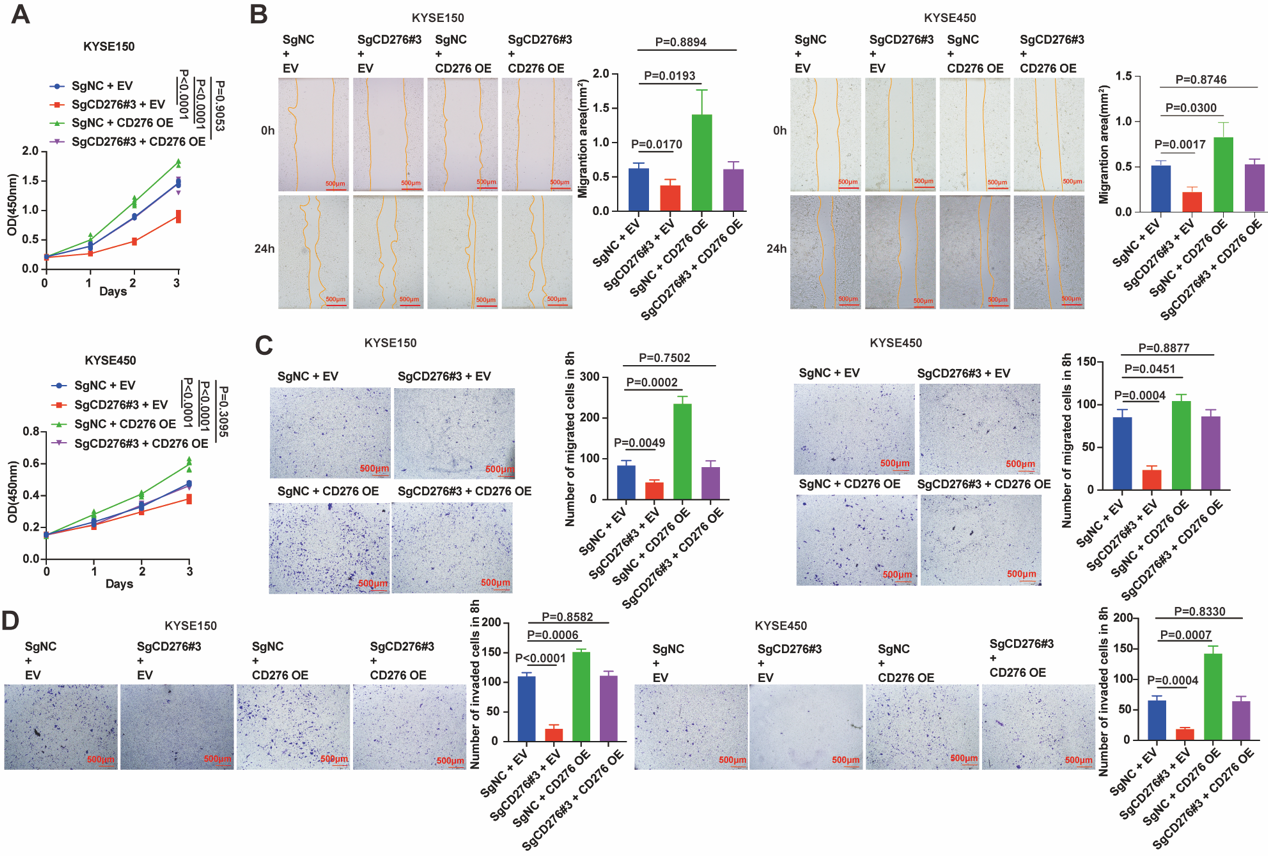
**

**A:** CCK8 proliferation assay showing that CD276 re expression restored the proliferation rate suppressed by CD276 knockout in KYSE150 and KYSE450 cells. **B：**Representative wound healing images and quantification demonstrating that CD276 re expression reinstated the migratory ability impaired by CD276 loss (scale bar = 500 μm).**C:** Transwell migration assays confirming that CD276 overexpression reversed the migration defect of CD276 deficient cells (scale bar = 500 μm). **D:** Matrigel invasion assays confirming that CD276 restoration rescued the reduced invasive potential induced by CD276 knockout (scale bar = 500 μm). Quantifications were performed using ImageJ. n = 3 per group. P values were calculated using Student’s t-test. All quantitative data are presented as mean ± SD with 95% CIs.

**Figure S3:** **SLC1A5 inhibitor V9302 inhibits CD276 expression by inducing autophagy in ESCC cells.**

**
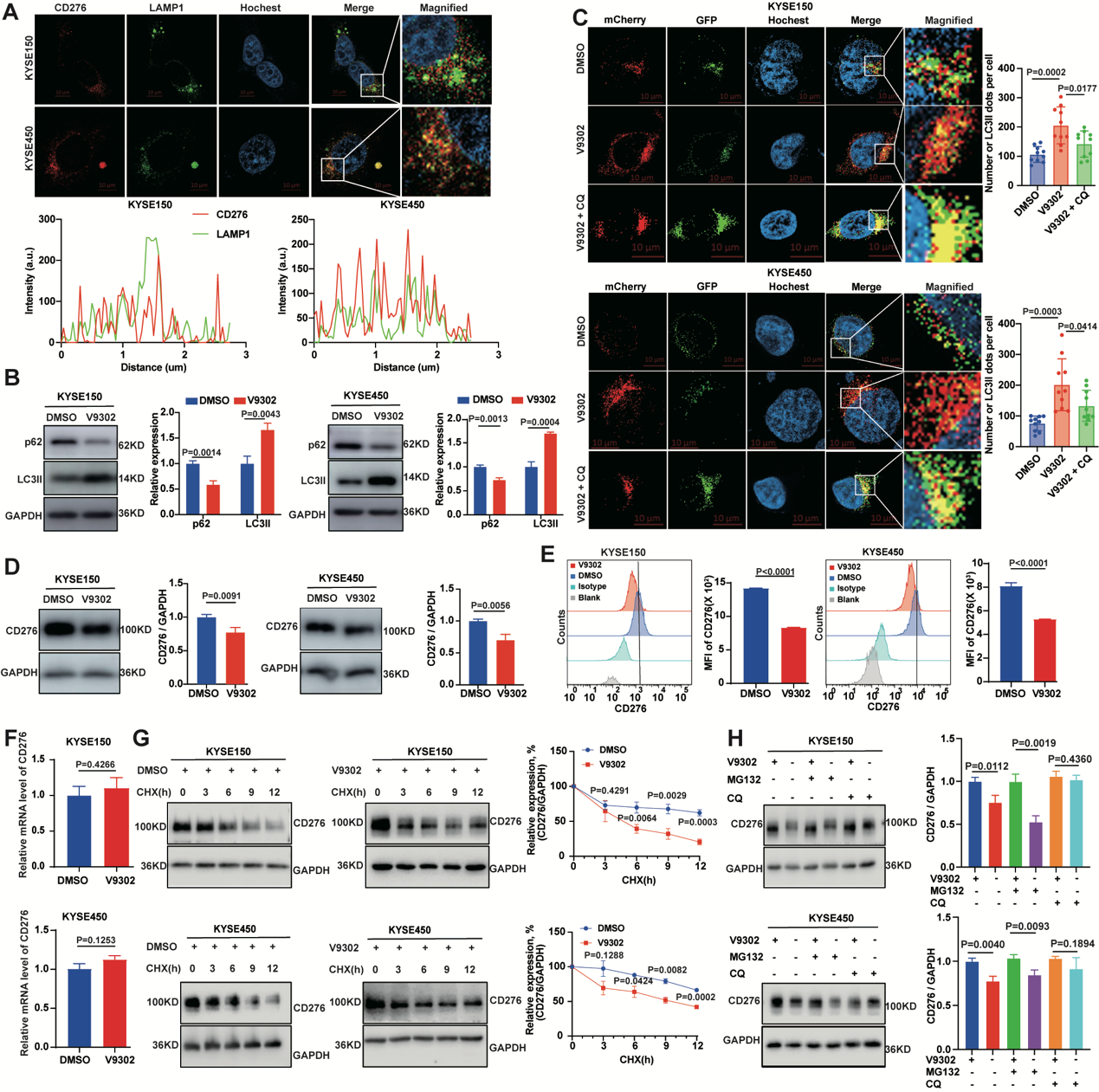
**

**A:** Co-localization of CD276 and LAMP1 was examined by confocal microscopy in KYSE150 and KYSE450 cells after glutamine deprivation to evaluate lysosomal localization of CD276. Fluorescence intensity profiles along indicated lines show the degree of CD276-LAMP1 overlap, indicating enhanced co-localization upon V9302 treatment (scale bar = 10 μm). **B:** Western blot analysis was performed to detect p62 and LC3II levels in KYSE150 and KYSE450 cells treated with V9302 (10 μM, 24 h). Band intensities were quantified using ImageJ and normalized to GAPDH. **C:** Autophagic flux assays were performed by infecting KYSE150 and KYSE450 cells with an mCherry-GFP-LC3II lentiviral vector, followed by V9302 treatment. Cells were imaged by confocal microscopy, and mCherry-GFP positive puncta were quantified (scale bar = 500 μm). **D:** CD276 expression in V9302 treated KYSE150 and KYSE450 cells was assessed by western blot. **E:** CD276 expression was assessed by flow cytometry in KYSE150 and KYSE450 cells after V9302 treatment. **F:** CD276 mRNA levels in KYSE150 and KYSE450 cells, either untreated or treated with V9302, were measured by RT-qPCR. Data are presented as relative expression (treated vs. untreated) using the 2^−ΔΔCt^ method. **G:** KYSE150 and KYSE450 cells were treated with V9302, followed by CHX at the indicated time points. CD276 protein levels was quantified using ImageJ. CD276 protein levels were quantified using ImageJ and normalized to GAPDH. **H:** CD276 expression in KYSE150 and KYSE450 cells treated with V9302, followed by MG132 (10 μM, 12 h) or CQ (25 μM, 12 h), was assessed by western blot. Band intensities were analyzed by ImageJ and normalized to GAPDH. n = 3 per group. P values were calculated using Student’s t-test. All quantitative data are presented as mean ± SD with 95% CIs.

**Figure S4. Flow cytometry gating strategy and control plots for CD276 and ROS detection in ESCC cells.**

**
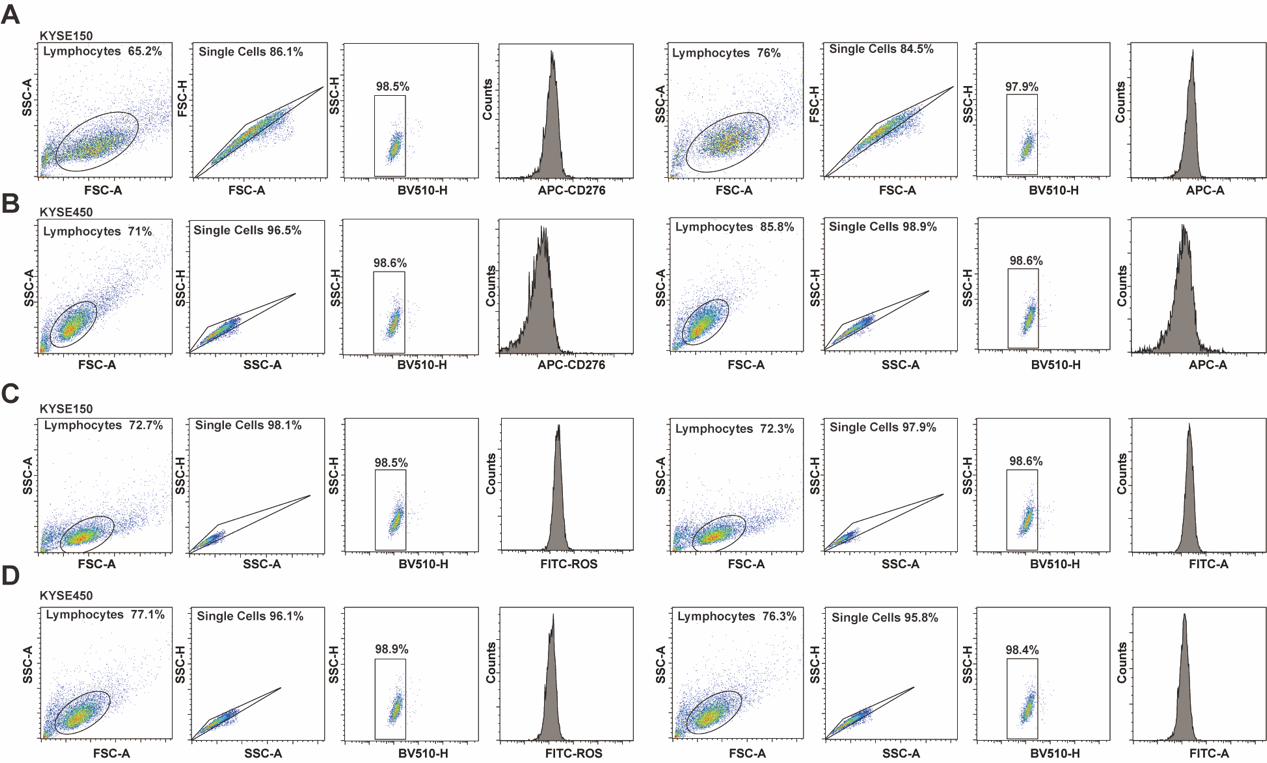
**

**(A–B)** Flow cytometry gating and detection of CD276 surface expression in KYSE150 **(A)** and KYSE450 **(B)** cells: Debris exclusion: Cells were first gated by forward scatter area (FSC-A) vs side scatter area (SSC-A) to remove cell debris (first panel, “Lymphocytes” gate represents the main cell population); Single-cell gating: FSC height (FSC-H) vs FSC-A plots were used to exclude cell doublets/aggregates (second panel, “Single Cells” gate); Viability gating: BV605 channel (Zombie Violet staining) was used to select Zombie Violet-negative cells (third panel, “BV605-H” gate), representing viable cells; CD276 detection: Histogram plots show APC-CD276 fluorescence intensity (gray curve) of viable cells, with the corresponding isotype control as the negative reference (fourth panel). **(C–D)** Flow cytometry gating and detection of intracellular ROS levels in KYSE150 **(C)** and KYSE450 **(D)** cells: Debris exclusion: Cells were gated by FSC-A vs SSC-A to remove debris (first panel, “Lymphocytes” gate); Single-cell gating: SSC height (SSC-H) vs SSC-A plots were used to exclude cell doublets/aggregates (second panel, “Single Cells” gate); Viability gating: BV605 channel (Zombie Violet staining) was used to select Zombie Violet-negative viable cells (third panel, “BV605-H” gate); ROS detection: Histogram plots show FITC fluorescence intensity (reflecting intracellular ROS levels) of viable cells, with unstained cells as the negative control (fourth panel).

**Figure S5.** **SLC1A5 knockdown inhibits the proliferation and metastasis of ESCC cells.**

**
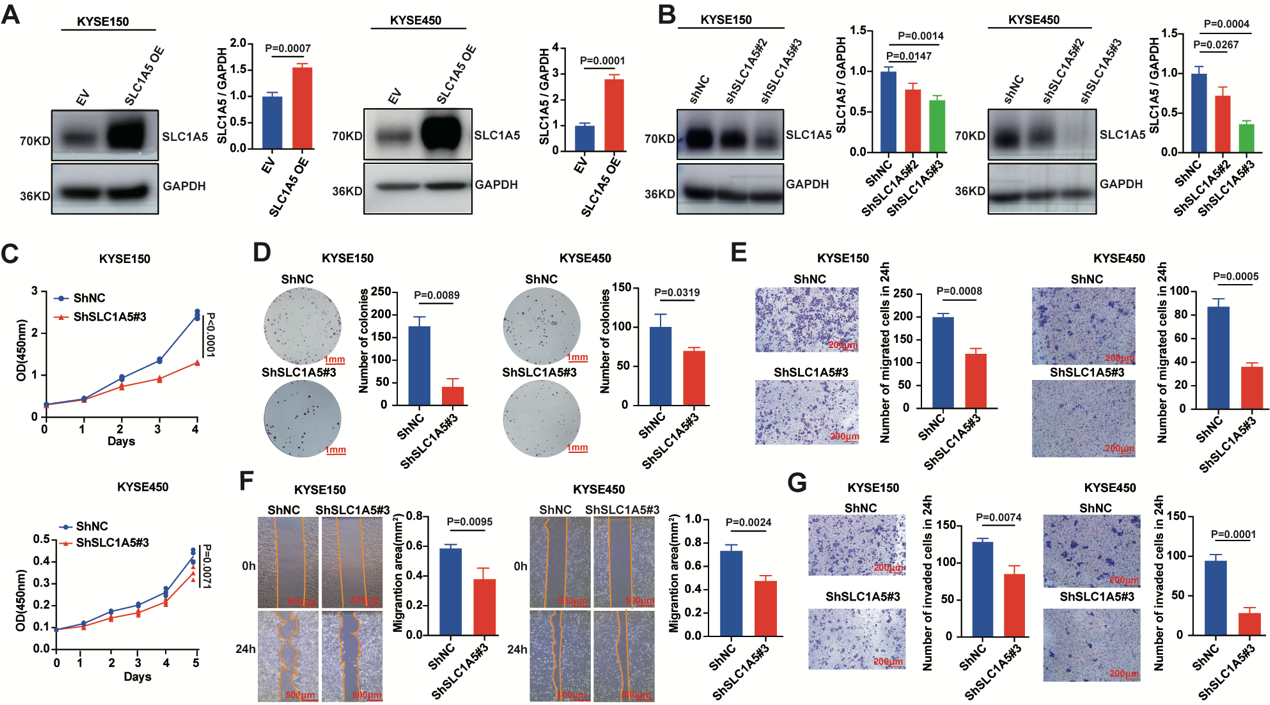
**

**A:** The efficiency of SLC1A5 overexpression ​​in​​ KYSE150 and KYSE450 cells ​​was validated by western blot. Densitometric values were obtained using ImageJ, normalized to GAPDH**. B:** The efficiency of SLC1A5 knockdown in KYSE150 and KYSE450 cells was confirmed by western blot. **C:** The effect of SLC1A5 knockdown (ShSLC1A5) on the proliferation of KYSE150 and KYSE450 cells was determined by CCK-8 assay. ShNC transfected cells as negative controls. **D:** The effect of SLC1A5 knockdown on proliferation was evaluated by colony formation assays in KYSE150 and KYSE450 cells. (scale bar = 1mm). **E:** Cell migration after SLC1A5 knockdown was assessed by transwell assays; representative images and quantification are shown. (scale bar = 200 μm). **F:** The effect of SLC1A5 knockdown on cell migration was evaluated by wound healing assays representative images and quantification are shown. (scale bar = 500 μm). **G:** Cell invasion after SLC1A5 knockdown was assessed by ​​Matrigel-coated transwell assays​​; representative images and quantification are shown. (scale bar = 200 μm). All images were quantified with ImageJ software. n = 3 per group. P values were calculated using Student’s t-test. All quantitative data are presented as mean ± SD with 95% CIs.

**Figure S6. Inhibition of glutamine metabolism inhibits the proliferation and metastasis of ESCC cells.**

**
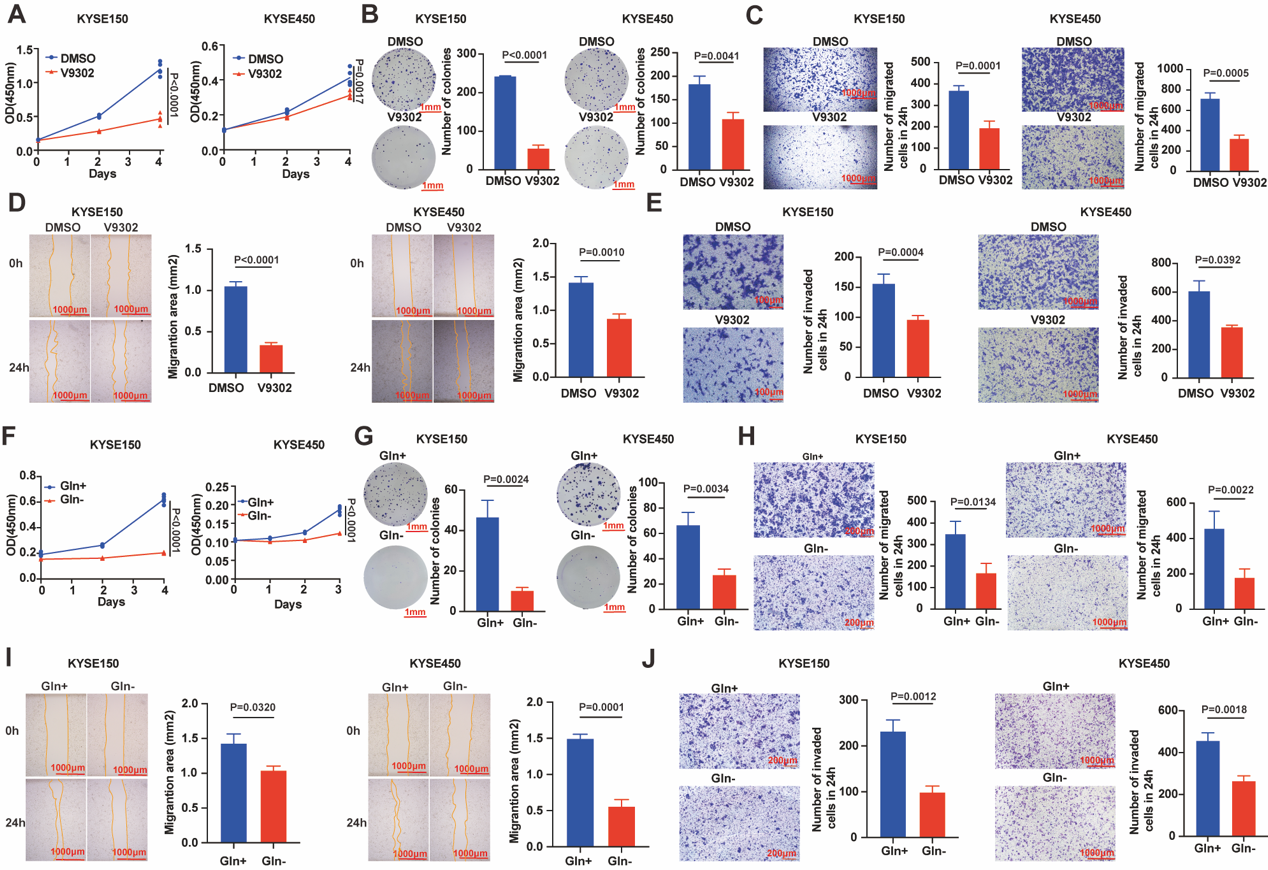
**

**A:** The effect of V9302 on the proliferation of KYSE150 and KYSE450 cells was determined by CCK-8 assay. **B:** Colony formation assays were used to ​​assess the effect of V9302 on colony formation in KYSE150 and KYSE450 cells. (scale bar = 1mm). **C:** Cell migration after V9302 treatment was assessed by transwell assays. (scale bar = 100 μm). **D:** Representative wound-healing images show altered migration in V9302-treated KYSE150 and KYSE450 cells. (scale bar = 1000 μm). **E:** Cell invasion after V9302 treatment was assessed by Matrigel-coated transwell assays. (scale bar = 100 μm). **F:** Cell growth curves ​​show​​ the proliferation of KYSE150 and KYSE450 cells cultured in glutamine-deprived (Gln−) or glutamine-supplemented (Gln+) medium. **G:** The impact of glutamine deprivation on clonogenic potential in KYSE150 and KYSE450 cells ​​was evaluated​​ by colony formation assays. (scale bar = 1mm). **H:** Cell migration after glutamine deprivation was assessed by transwell assays (scale bar = 200μm (KYSE150)). (scale bar = 1000μm (KYSE450)). **I:** Wound healing assays illustrate changes in cell motility following glutamine deprivation. (scale bar = 1000μm). **J:** Invasive potential of KYSE150 and KYSE450 cells was examined by transwell invasion assays under glutamine-deprived conditions​​. (scale bar = 200μm (KYSE150)). (scale bar = 1000μm (KYSE450)). All images were quantified with ImageJ software. n = 3 per group. P values were calculated using Student’s t-test. All quantitative data are presented as mean ± SD with 95% CIs.

**Figure S7:** **SLC1A5 knockout promotes CD276 degradation by inducing autophagy.**

**
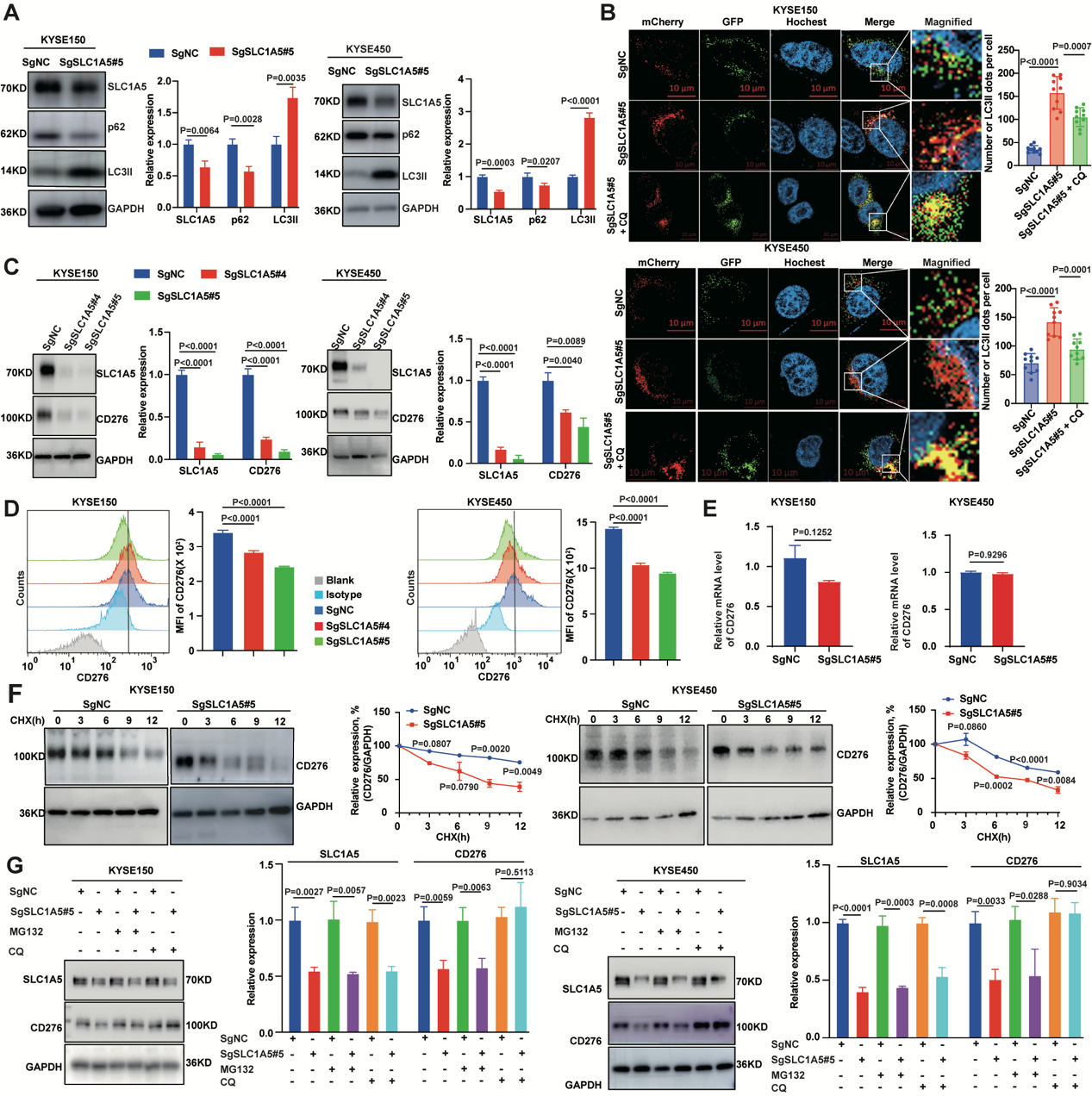
**

**A:**Western blot revealed autophagic marker dynamics (p62, LC3II) in SLC1A5 knockout KYSE150 and KYSE450 cells. Densitometric analysis of Western blot results normalized to GAPDH is presented on the right. **B:**Autophagic flux ​​was assessed​​ by infecting cells with​​ an mCherry-GFP-LC3II​​ lentiviral vector​​, followed by confocal microscope; the number of mCherry and GFP puncta was quantified. (scale bar = 10μm). **C:**Western blot confirmed ​​SLC1A5 knockout efficiency​​ and ​​its effect on CD276 regulation​​ in KYSE150 and KYSE450 cells. Quantification of band intensity relative to GAPDH is shown on the right. **D:** CD276 protein expression ​​was quantified by flow cytometry​​ in SLC1A5 knockout KYSE150 and KYSE450 cells. **E:** CD276 mRNA levels ​​were measured by RT-qPCR​​ after SLC1A5 knockout in KYSE150 and KYSE450 cells. **F:**CD276 protein stability ​​was assessed by western blot​​ in SLC1A5 knockout cells treated with CHX​​. Band intensities were quantified using ImageJ and normalized to GAPDH. **G:**CD276 degradation pathway ​​was examined by Western blot in SLC1A5 knockout cells treated with MG132 or ​​CQ​. Densitometric values were measured using ImageJ and normalized to GAPDH; quantified results are shown on the right. n = 3 per group. P values were calculated using Student’s t-test. All quantitative data are presented as mean ± SD with 95% CIs.

**Figure S8:** **Inhibition of glutamine metabolism promotes ROS-dependent autophagic degradation of CD276.**

**
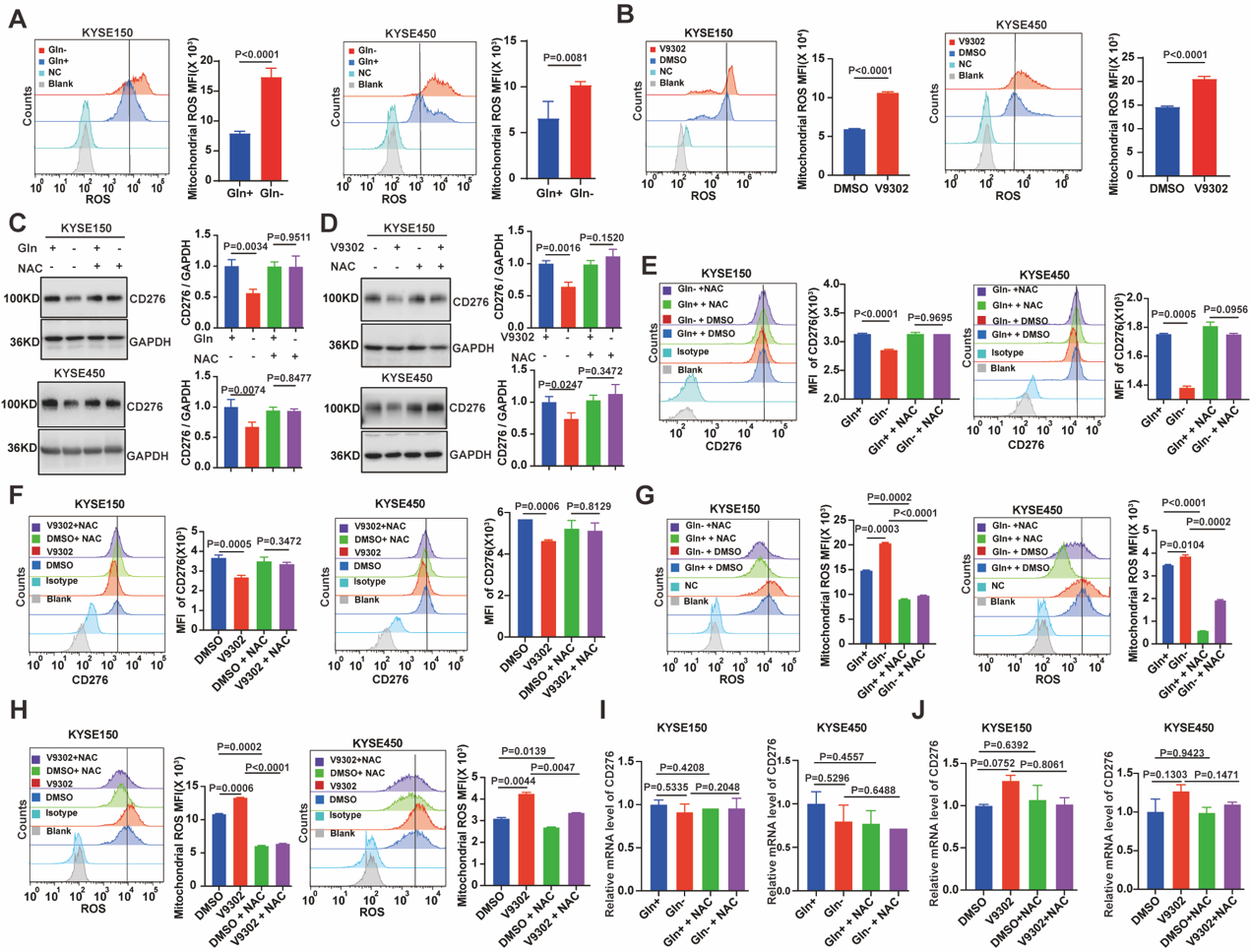
**

**A:** Mitochondrial ROS levels were measured by flow cytometry in KYSE150 and KYSE450 cells under glutamine deprivation. **B:** Flow cytometry revealed mitochondrial ROS accumulation in V9302-treated KYSE150 and KYSE450 cells. **C:** CD276 protein levels were assessed by Western blot in KYSE150 and KYSE450 cells under glutamine deprivation ± NAC (2.5 mM). Densitometric quantification of CD276 normalized to GAPDH is shown on the right. **D:** CD276 protein levels were evaluated by Western blot in KYSE150 and KYSE450 cells treated with V9302 ± NAC. Densitometric analysis of CD276 band intensity relative to GAPDH is shown on the right. **E:** Flow cytometry revealed CD276 expression in glutamine deprived KYSE150 and KYSE450 cells ± NAC. **F:** CD276 surface expression was measured by flow cytometry in V9302-treated KYSE150 and KYSE450 cells ± NAC. **G:** Mitochondrial ROS levels were measured by flow cytometry in glutamine deprived KYSE150 and KYSE450 cells ± NAC. **H:** Mitochondrial ROS levels were monitored by flow cytometry in V9302-treated KYSE150 and KYSE450 cells ± NAC. **I:** RT-qPCR analysis quantified CD276 mRNA levels in glutamine-deprived KYSE150 and KYSE450 cells ± NAC. **J:** CD276 mRNA levels were quantified by RT-qPCR in V9302-treated KYSE150 and KYSE450 cells ± NAC. n = 3 per group. P values were calculated using Student’s t-test. All quantitative data are presented as mean ± SD with 95% CIs.

**Figure S9. Combination therapy targeting glutamine metabolism and CD276 in ESCC *in vitro*.**

**
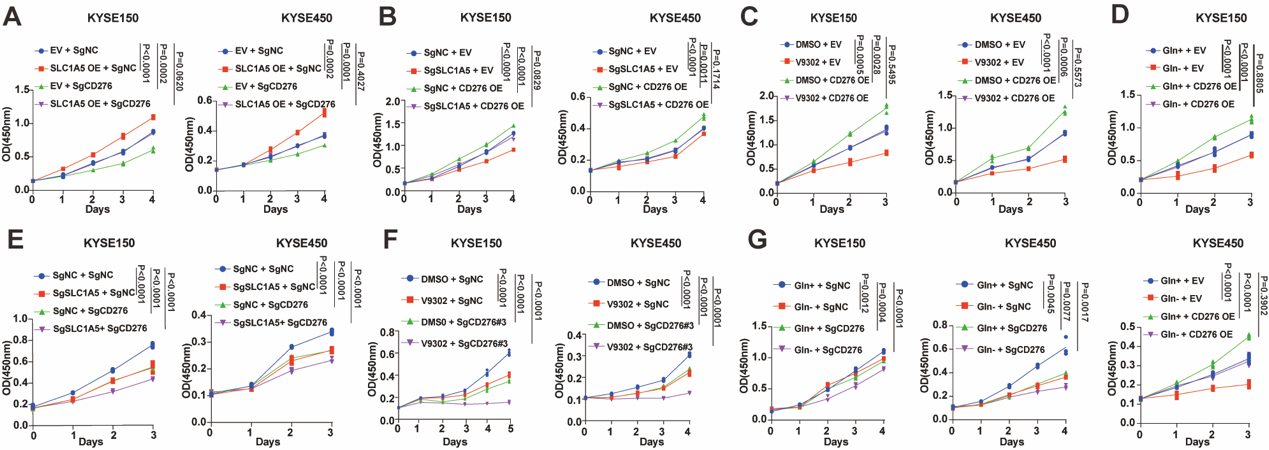
**

CCK-8 proliferation assays following combination treatment targeting glutamine metabolism and CD276 in ESCC cells: **A:**SLC1A5 overexpression in CD276 knockout cells; **B:**SLC1A5 knockout in CD276 overexpressing cells; **C:** V9302 treatment of CD276 overexpressing cells; **D:**Glutamine deprivation in CD276 overexpressing cells; **E:** SLC1A5 knockout in CD276 knockout cells; **F:**V9302 treatment of CD276 knockout cells; **G:** Glutamine deprivation in CD276 knockout cells. n = 3 per group. P values were calculated using Student’s t-test. All quantitative data are presented as mean ± SD with 95% CIs.

**Figure S10. V9302 regulate CD276 protein expression across distinct treatment groups.**


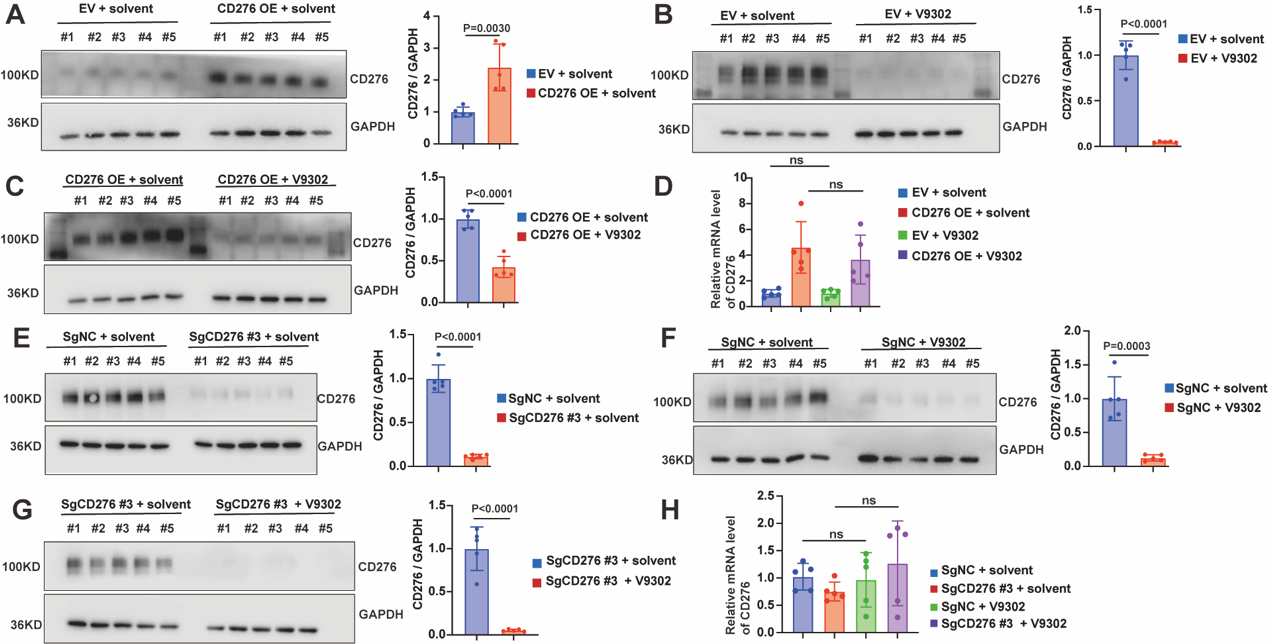


**A:** Western blot images and quantitative analysis of CD276 protein in EV + solvent and CD276 OE + solvent groups. **B:** Western blot images and quantification of CD276 in EV + solvent and EV + V9302 groups. **C:** Western blot images and quantitative analysis of CD276 in CD276 OE + solvent and CD276 OE + V9302 groups. **D:** RT-qPCR analysis quantified CD276 mRNA levels across four groups (EV + solvent, CD276 OE + solvent, EV + V9302, CD276 OE + V9302). **E:** Western blot images and quantification of CD276 in SgNC + solvent and SgCD276 #3 + solvent groups. **F:** Western blot images and quantitative analysis of CD276 in SgNC + solvent and SgNC + V9302 groups. **G:** Western blot images and quantification of CD276 in SgCD276 #3 + solvent and SgCD276 #3 + V9302 groups. **H:** RT-qPCR analysis quantified CD276 mRNA levels across four groups (SgNC + solvent, SgCD276 #3 + solvent, SgNC + V9302, SgCD276 #3 + V9302).
